# Downregulation of CPSF6 leads to global mRNA 3’ UTR shortening and enhanced antiviral immune responses

**DOI:** 10.1371/journal.ppat.1012061

**Published:** 2024-02-28

**Authors:** Yong Ge, Jingrong Huang, Rong Chen, Yonggui Fu, Tao Ling, Xin Ou, Xiaohui Rong, Youxiang Cheng, Yi Lin, Fengyi Zhou, Chuanjian Lu, Shaochun Yuan, Anlong Xu

**Affiliations:** 1 Guangdong Province Key Laboratory of Pharmaceutical Functional Genes, MOE Key Laboratory of Gene Function and Regulation, State Key Laboratory of Biocontrol, School of Life Sciences, Sun Yat-Sen University, Guangzhou, China; 2 Southern Marine Science and Engineering Guangdong Laboratory (Zhuhai), Zhuhai, China; 3 The Second Clinical College of Guangzhou University of Chinese Medicine, Guangzhou, China; 4 State Key Laboratory of Dampness Syndrome of Chinese Medicine, Guangdong-Hong Kong-Macau Joint Lab on Chinese Medicine and Immune Disease Research, The Second Affiliated Hospital of Guangzhou University of Chinese Medicine, Guangdong Provincial Academy of Chinese Medical Sciences, Guangzhou, China; 5 School of Life Sciences, Beijing University of Chinese Medicine, Beijing, China; The University of Chicago, UNITED STATES

## Abstract

Alternative polyadenylation (APA) is a widespread mechanism of gene regulation that generates mRNA isoforms with alternative 3’ untranslated regions (3’ UTRs). Our previous study has revealed the global 3’ UTR shortening of host mRNAs through APA upon viral infection. However, how the dynamic changes in the APA landscape occur upon viral infection remains largely unknown. Here we further found that, the reduced protein abundance of CPSF6, one of the core 3’ processing factors, promotes the usage of proximal poly(A) sites (pPASs) of many immune related genes in macrophages and fibroblasts upon viral infection. Shortening of the 3’ UTR of these transcripts may improve their mRNA stability and translation efficiency, leading to the promotion of type I IFN (IFN-I) signalling-based antiviral immune responses. In addition, dysregulated expression of CPSF6 is also observed in many immune related physiological and pathological conditions, especially in various infections and cancers. Thus, the global APA dynamics of immune genes regulated by CPSF6, can fine-tune the antiviral response as well as the responses to other cellular stresses to maintain the tissue homeostasis, which may represent a novel regulatory mechanism for antiviral immunity.

## Author summary

Innate antiviral immunity endows the host with a first line of defense against viral infection. Efficient regulatory manners are needed to modulate the strength and duration of the response and recover initial state. APA, a widespread gene regulatory mechanism in eukaryotes that generates messenger RNA isoforms with different 3’ UTR lengths, has emerged as a key mechanistic step in the regulation of the cellular response to environmental stimuli or perturbation, including viral infection. The global 3’ UTR shortening of mRNAs through APA that occurs during viral infection, as described in our previous study, may represent an important yet poorly understood regulatory mechanism of antiviral immune responses. Here, we further find that the reduced protein expression of CPSF6, a core 3’ processing factor, effectively contributes to the global 3’ UTR shortening of many immune related genes after viral infection, which improves their mRNA stability and translation efficiency and promote the establishment of antiviral defense. Hence, the global 3’ UTR shortening caused by reduced CPSF6 during viral infection orchestrates the cellular antiviral capacity to inhibit viral infection. These findings suggest that CPSF6-mediated APA is a smart regulator of antiviral immune responses, opening up new research ideas for the treatment of viral infections and related diseases.

## Introduction

Alternative polyadenylation (APA) is a widespread phenomenon occurring during eukaryotic mRNA maturation, generating mRNAs with alternative 3’ UTR ends [1,2]. More than half of mammalian mRNAs contain multiple polyadenylation sites (PASs), and APA in the 3’ UTR (3’ UTR-APA) enables the production of distinct mRNA isoforms with the same coding capacity but different 3’ UTR lengths. Since the 3’ UTR may act as a scaffold for protein–RNA interactions and contains many microRNA (miRNA) response elements, AU-rich elements (AREs), GU-rich elements (GREs), and Alu elements [3–6], 3’ UTR-APA can affect the stability, localization and translation efficiency of target mRNAs and is thus commonly involved in distinct biological processes including cell growth, differentiation, stem cell renewal, and the immune response [7]. For example, transcripts expressed in the testis tend to have shorter 3’ UTRs than those expressed in other tissues, while transcripts with longer 3’ UTRs are abundant in the nervous system [8,9]. In addition, cancer cells [10] and proliferating cells [11] tend to use proximal PASs, in contrast to differentiated cells and developing embryonic cells [12,13]. Biased usage of the proximal PAS and widespread shortening of 3’ UTRs have also been found in the stimulated or activated B lymphocytes, T cells, and monocytes [11, 14, 15].

In addition to the above roles, APA is deeply involved in the acute immune response, especially in the defense against viral infection [16]. Upon viral infection, pattern recognition receptors (PRRs) such as RIG-I, cGAS and TLR3 can recognize virus-derived pathogen-associated molecular patterns (PAMPs) and then trigger the activation of IRFs and NF-κB via adaptor proteins such as MAVS, STING and TRIF, leading to the transcriptional induction of type I/III IFNs (IFN-I/-III) and proinflammatory cytokines [17–19]. To efficiently initiate the antiviral immune response and maintain the tissue homeostasis [20,21], the plasticity and complexity of antiviral immunity should be coordinately and temporally regulated at multiple levels, including the transcriptional or translational switches [22,23], and the posttranscriptional or posttranslational modifications [24,25]. Recently, genome-wide APA dynamics were revealed upon viral infection in multiple organisms. For example, extensive dynamic changes in APA and the use of atypical polyadenylate signalling were identified in JEG3 cells infected with Zika virus (ZIKV) [26]. Analysis of the RNA-Seq data from PBMCs of COVID-19 patients showed that many genes related to host response to COVID-19, such as neutrophil activation, MAPK cascade signalling, and regulation of cytokine production as well as IFN-γ secretion, underwent dynamic APA changes [27]. The infection of HSV-1 also leads to the use of more upstream poly (A) sites in many host genes, including many intron PASs (Intronic PASs, IPAs) [28]. A dynamic shortened landscape of tandem 3’ UTRs in VSV-infected macrophages, which promotes the establishment of macrophage antiviral immune status in conjunction with gene transcription regulation, was also identified in our previous study [29]. In addition to fine-tuning the host antiviral immune response, viruses selectively disrupt host 3’-end processing to cause widespread host shutoff to possibly suppress the cellular antiviral response [28,30], suggesting that APA can orchestrate the antiviral complexity in aspects related to both the host and virus.

APA processing is regulated by alternatively stimulating or suppressing cleavage and poly(A) addition, depending on the level of core 3’ processing factors and the RNA sequences surrounding the potential cleavage sites [31]. The core components of the mammalian APA machinery include dozens of polypeptides, most of which exist in multisubunit subcomplexes, such as cleavage and polyadenylation specificity factor (CPSF), cleavage stimulation factor (CstF), cleavage factor Im (CFIm) and CFIIm [32]. Alterations in the abundance or activity of these core 3’ processing factors have been found to perform global effects on APA in a variety of biological processes [33,34], as well as in some immune states. For example, infection with human cytomegalovirus (HCMV) can induce upregulation of the host polyadenylation factor CPEB1, resulting in shorter 3’ UTRs and longer poly (A) tail lengths of genes in host cells [35]. Overexpression of CstF64, a 3’ processing factor and known regulator of polyadenylation efficiency, increases the usage of promoter-proximal poly(A) sites and promotes macrophage differentiation without induction [36]. The 3’ end processing factor NUDT21 limits expression of CD19 in B-cell progenitor acute lymphoblastic leukemia (B-ALL) cells by regulating CD19 mRNA polyadenylation [37]. In VSV-infected macrophages, dynamic changes in the expression of many 3’ processing factors were identified, indicating that regulation of the expression of 3’ processing factors may be one of the reasons underlying genome-wide APA shortening upon viral infection [29]. In this study, after identifying the reduced expression of CPSF6, a subunit of the CFIm, during viral infection, we further showed that knocking down of CPSF6 promotes the activation of IFN-I signalling and inhibits viral replication. Using the IVT-SAPAS-Seq (*in vitro* transcription-sequencing APA sites) [38, 39] to identify genes regulated by CPSF6-mediated APA upon viral infection, we then revealed the novel roles of CPSF6 in fine-tuning the antiviral immune response, which not only explains how the landscape of shortened 3’ UTRs is mediated upon viral infection, but also opens a new line of investigation into the treatment of viral infections and related diseases.

## Results

### CPSF6 protein expression is decreased in virus-infected cells

**Fig 1:**
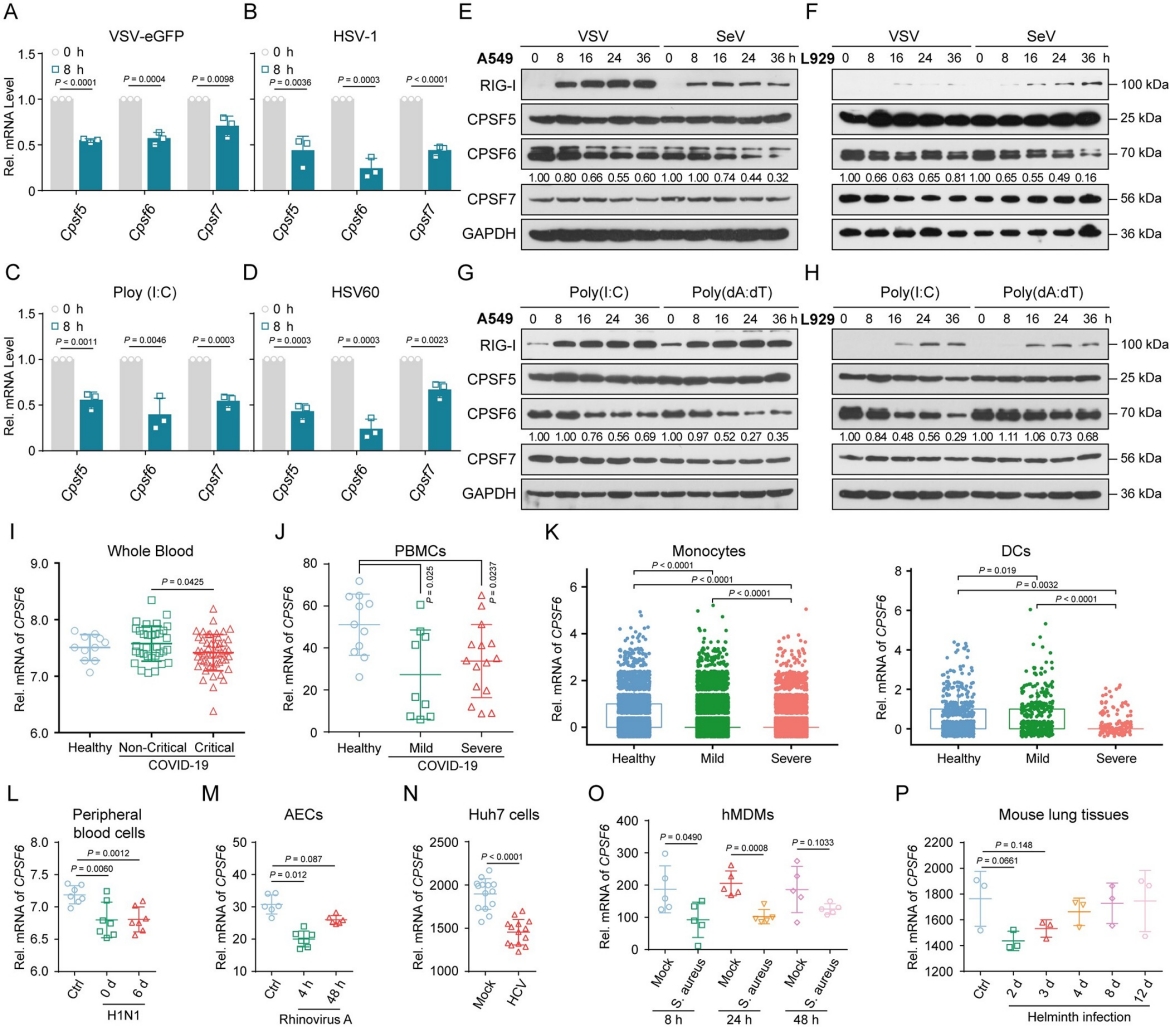
CPSF6 protein expression is decreased in virus-infected cells. (**A**-**D**) Mouse BMDMs were challenged with VSV (**A**), HSV-1 (**B**) poly (I:C) (**C**) or HSV60 (**D**) for 12h, and the mRNA levels of core 3’ processing factors were analyzed by qRT–PCR. (n = 3 replicates) (**E**, **F**) Immunoblot analyses of CFIm complex expression in A549 (**E**) and L929 (**F**) cells infected with VSV or SeV at indicated time points. (**G**, **H**) Immunoblot analyses of CFIm complex expression in A549 (**G**) and L929 (**H**) cells stimulated with poly (I:C) or Poly (dA:dT) at indicated time points. The expression of RIG-I indicates that viral infection or analogue stimulation has successfully activated the antiviral signaling of cells. (**I**-**L**) The relative mRNA expression of *CPSF6* in whole blood (**I**), PBMCs (**J**), monocytes and DCs (**K**) from patients with COVID-19 or in peripheral blood cells (**L**) from patients with influenza are shown. (**M-P**) The relative mRNA expression of *CPSF6* in RV-A16-infected AECs (**M**), HCV-infected Huh7 cells (**N**), *S*. *aureus*-infected hMDMs (**O**) and helminth-infected mouse lung tissues (**P**) are shown. Data are representative of three independent experiments, with one representative shown in (**E**-**H**). The values represent mean ± SD with individual measurements overlaid as dots, statistical analysis was performed using a two-tailed Student’s *t*-test or Mann–Whitney U test in (**A**-**D**) and (**I**-**P**).

Knockdown of CPSF5 (CFIm25) or CPSF6 (CFIm68), two key subunits of the CFIm tetramer leads to a widespread increase in the use of proximal PASs (pPASs) [40], making this complex a key candidate to reveal the genome-wide landscape of shortened tandem 3’ UTRs upon viral infection. To explore the mechanism of the widespread dynamic changes in APA in host cells after viral infection, we first tested the change in the mRNA expression of CPSF6 and the other two members of CFIm upon viral infection or nucleic acid analogue stimulation. The data indicated that all the members of the CFIm complex exhibited decreased expression upon infection with VSV (vesicular stomatitis virus) or HSV-1 (herpes simplex virus 1) (Fig 1A and 1B) or stimulation with the nucleic acid analogue Poly (I:C) LMW (low molecular weight) or HSV60 (synthetic herpes simplex virus 1 DNA analogue) in BMDMs (mouse bone marrow-derived macrophages) (Fig 1C and 1D). Interestingly, the protein expression of CPSF6 was significantly decreased after viral infection or analogue simulation, accompanied by a decreased RNA abundance, while CPSF5 and CPSF7 (CFIm59) showed no significant changes (S1A and S1B Fig). Similar results were obtained in VSV-eGFP- or SeV (Sendai virus)-infected, or in Poly (I:C)- or Poly (dA:dT)-stimulated A549 and L929 cells (Fig 1E–1H). These data collectively suggested that the protein abundance of CPSF6 protein is reduced in multiple virus-infected cells.

To confirm this observation, we conducted statistical analyses of previously published gene expression datasets (GSE213313/149689/150728/236651/27131/226071/20948/13670/3414) and found that the mRNA expression of CPSF6 was significantly lower in SARS-CoV-2- (Fig 1I–1K) or influenza (H1N1)-infected patients (Fig 1L), rhinovirus A16 (RV-A16)-infected primary human airway epithelial cells (AECs) (Fig 1M) and hepatitis C virus (HCV, JFH-1)-infected Huh7 cells (Fig 1N). Not only viral infections, but also bacterial or helminthic infections can lead to downregulation of CPSF6 (Fig 1O and 1P). All these data indicated that downregulated expression of CPSF6 is a common phenomenon in the immune response to infection, implying that CPSF6 may play an important regulatory role in pathogen infection, especially viral infection.

### CPSF6 deficiency inhibits virus replication

**Fig 2:**
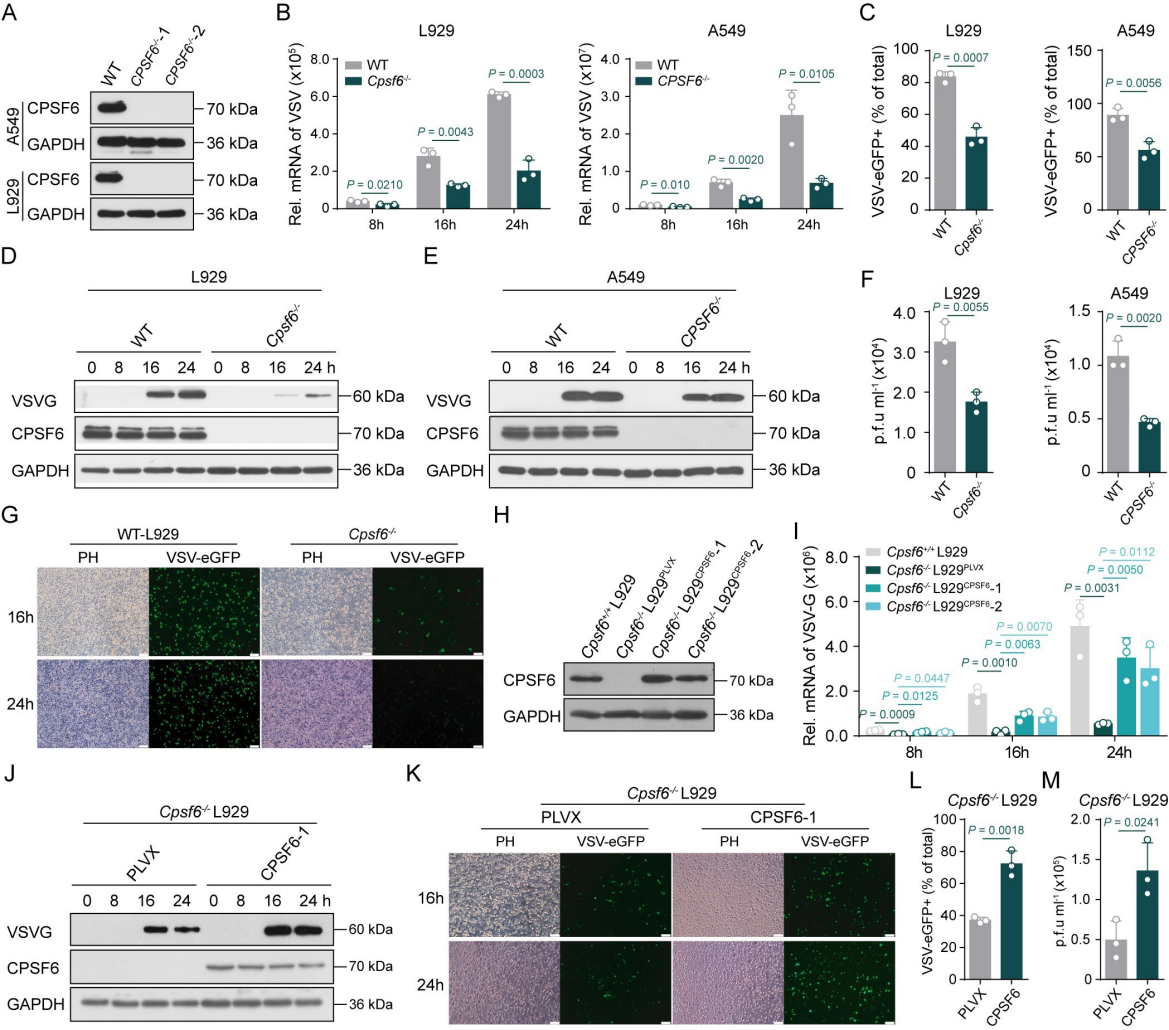
CPSF6 deficiency inhibits virus replication. **(A**) Immunoblot analyses knocking-out efficiency of CPSF6 in A549 and L929 cells. (**B**) qRT–PCR analyses of VSV mRNA abundance in WT and *CPSF6*^-/-^ A549 and L929 cells infected with VSV-eGFP at indicated time points. (n = 3 replicates) (**C**) FACS analyses of VSV replication in WT and *CPSF6*^-/-^ A549 and L929 cells infected with VSV-eGFP for 12 h. (n = 3 replicates) (**D**, **E**) Immunoblot analyses of VSVG protein expression in WT and *CPSF6*^-/-^ L929 (**D**) or A549 (**E**) cells infected with VSV-eGFP at indicated time points. (**F**) Plaque assays of VSV titers in cell supernatants from WT and *Cpsf6*^-/-^ L929 cells infected with VSV-eGFP for 12 h. (n = 3 replicates) (**G**) Fluorescence microscope analyses of GFP intensity in WT and *Cpsf6*^-/-^ L929 cells infected with VSV-eGFP for 16 or 24 h. Scale bar, 100 μm. (**H**) Immunoblot analyses the expression of CPSF6 in WT, *Cpsf6*^-/-^L929^PLVX^ and *Cpsf6*^-/-^ L929^*CPSF6*^ L929 cells. (**I**) qRT–PCR analyses of VSV mRNA abundance in WT, *Cpsf6*^-/-^L929^PLVX^ and *Cpsf6*^-/-^ L929^*CPSF6*^ L929 cells infected with VSV-eGFP at indicated time points. (n = 3 replicates) **(J**) Immunoblot analyses of VSVG protein expression in *Cpsf6*^-/-^L929^PLVX^ and *Cpsf6*^-/-^ L929^*CPSF6*^ L929 cells infected with VSV-eGFP at indicated time points. (**K**-**M**) Fluorescence microscope (**K**), FACS (**L**) and plaque assays (**M**) analyses of VSV expression in *Cpsf6*^-/-^L929^PLVX^ and *Cpsf6*^-/-^ L929^*CPSF6*^ L929 cells infected with VSV-eGFP at indicated time points. Scale bar, 100 μm. (n = 3 replicates). Data are representative of three independent experiments, with one representative shown in (**A**), (**D**), (**E**), (**H**) and (**J**). The values represent mean ± SD with individual measurements overlaid as dots, statistical analysis was performed using a two-tailed Student’s *t*-test in (**B**), (**C**), (**F**), (**I**), (**L**) and (**M**).

To explore the role of CPSF6 in antiviral immune response, we next evaluated the effect of CPSF6 on virus replication. First, we silenced the endogenous expression of CPSF6 in A549 and L929 cells (S2A Fig). Then, VSV-eGFP-infected cells were subjected to flow cytometry analyses. The results showed that knockdown of CPSF6 in A549 and L929 cells significantly inhibited the production of VSV virions (S2B Fig), accompanied by a lower VSV RNA abundance (S2C Fig). To further study the function of CPSF6 in regulating the antiviral response, we generated the *CPSF6*^*-/-*^ A549 and L929 cells using the CRISPR/Cas9 system (Fig 2A) and observed lower VSV replication and lower VSV RNA and protein abundances in these cells (Fig 2B–2E). Moreover, lower VSV titers in the cell culture supernatant (Fig 2F) and a significantly decreased VSV-eGFP abundance in *CPSF6*^*-/-*^ A549 and L929 cells were observed (Figs 2G and S2D). Furthermore, the replication of other types of viruses, such as the RNA virus SeV and DNA virus HSV-1, was also reduced in *Cpsf6*^*-/-*^ L929 cells (S2E–S2G Fig). To confirm the role of CPSF6, CPSF6-reconstituted L929 cells (hereinafter referred to as *Cpsf6*^*-/-*^ L929^*CPSF6*^ cells; Fig 2H) were established. Ectopic expression of CPSF6 resulted in increased VSV RNAs and proteins abundances (Fig 2I and 2J) compared with those in *Cpsf6*^*-/-*^ L929 cells. The fluorescence intensity of GFP and the load of VSV titers indicated that more VSV virions were produced in *Cpsf6*^*-/-*^ L929^CPSF6^ cells (Fig 2K–2M). In addition, qRT–PCR and immunoblot analyses indicated that the ectopic CPSF6 expression also increased HSV-1 replication (S2H and S2I Fig). All these data indicate that CPSF6 deficiency can inhibit the replication of both DNA and RNA viruses.

### Global 3’ UTR shortening caused by CPSF6 deficiency orchestrates the cellular antiviral capacity

**Fig 3:**
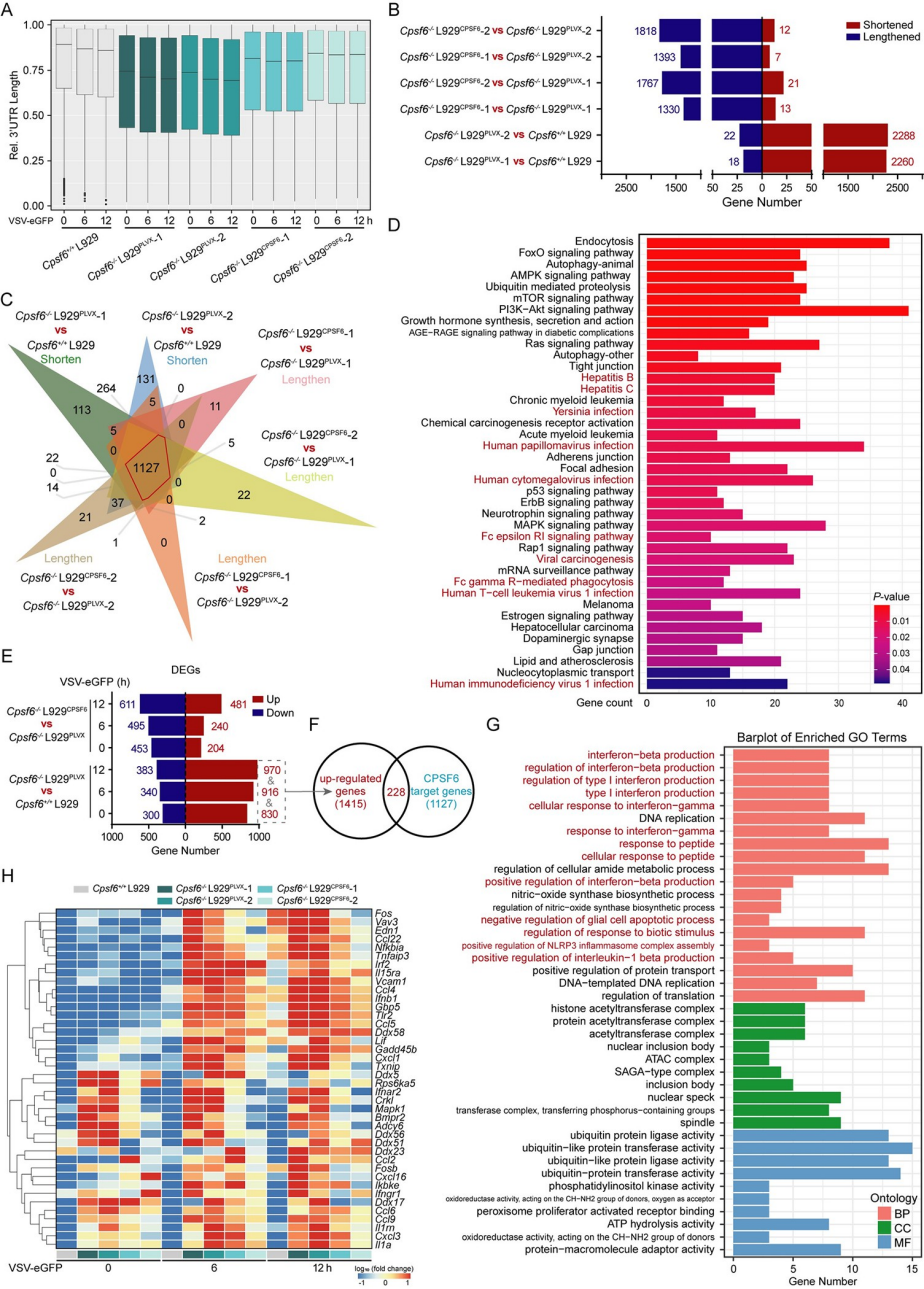
Global 3’ UTR shortening caused by CPSF6 deficiency orchestrates the cellular antiviral capacity. (**A**) Notched box plot of the weighted mean of the 3’ UTR length. For each gene with UTR-APA, the length of each 3′ UTR isoform was normalized to the longest 3’ UTR, and the weighted mean of 3’ UTR length was calculated. (**B**) The number of poly (A) site-switched genes in L929 cells at rest. (**C**), Venn diagram showing the overlap of genes from (**B**). (**D**) KEGG enrichment analysis of the overlapped transcripts presented in (**C**). (**E**) The number of differentially expressed genes (DEGs) in L929 cells upon VSV-eGFP infection. (**F**) Venn diagram showing the overlap of genes from CPSF6 target genes and up-regulated genes after the deletion of CPSF6. (**G**) GO analysis of the overlapped transcripts presented in (**F**). (**H**) The heatmap shows the mRNA abundance of immune-related genes from IVT-SAPAS data.

CPSF6 is a key component of the CFIm complex, and knocking down of CPSF6 leads to transcriptome-wide shortening of 3’ UTRs due to an increase in the use of pPASs [41]. Given the roles of CPSF6 in APA processing and in regulating viral infection as described above, we hypothesized that CPSF6 acts as an antiviral immune regulator by influencing APA and the 3’ UTR length. To test this hypothesis, we used the IVT-SAPAS method for transcriptome-wide APA profiling in VSV-eGFP-infected L929 cells with knockout or ectopic expression of CPSF6 [38,39]. After high-throughput sequencing, we found that more than 80% of the qualified reads were mapped to the annotated UCSC transcript ends database and Tian’s database (S3A Fig). Moreover, approximately 60% of polyadenylation signals are one of two canonical hexanucleotide polyadenylation signal sequences, the AATAAA or ATTAAA, most of which are located 10–30 bases upstream of the cleavage/polyadenylation site and specifically recognized by the CPSF [42] (S3B Fig). Consistent with previous studies, approximately 50% of genes contained more than one poly(A) site (S3C Fig). Consistent with our previous observation [29], the average 3’ UTR length in cells was decreased after viral infection and decreased even more upon CPSF6 deficiency (Fig 3A), further demonstrating the priority usage of proximal PASs in the absence of CPSF6. After statistical analysis, we found that more than 2000 genes showed shortened 3’ UTRs in CPSF6-deficient cells at rest and after viral infection, while the 3’ UTRs of approximately 1500 genes became longer when CPSF6 was ectopically expressed in CPSF6-deficient cells (Figs 3B, S3D and S3E). Subsequently, we defined the genes with shorter 3’ UTRs upon CPSF6 deficiency and longer 3’ UTRs upon CPSF6 restoration as the target genes of CPSF6, and finally identified 1127 target genes (Fig 3C), most of which are related to virus infection, autophagy, signal transduction, metabolism and cell proliferation, as determined using Kyoto Encyclopedia of Genes and Genomes (KEGG) pathway enrichment analysis (Fig 3D).

In addition to measuring tandem 3’ UTR lengths, we can also use our IVT-SAPAS data to quantify mRNA expression by totaling the reads mapped to the 3’ UTR of a given gene. We found that more transcripts were profoundly upregulated (>3 fold, FDR-adjusted P<0.01, Fisher’s exact test) in *Cpsf6*^*-/-*^ L929 cells than in WT cells with or without viral infection, while the ectopic expression of CPSF6 inhibited this upregulation (Fig 3E). Next, we identified 228 CPSF6 target genes among the upregulated genes upon CPSF6 deficiency (Fig 3F). Gene Ontology (GO) analysis further indicated that the terms interferon production and regulation, stress response, apoptosis and inflammasome assembly (Fig 3G), which are closely related to host response and resistance to invading pathogens, were significantly enriched in these genes. Along with the global 3’ UTR shortening, significant upregulation of a large number of immune-related genes was observed in CPSF6-deficient L929 cells after viral infection compared with the controls; in contrast, ectopic expression of CPSF6 in *Cpsf6*^*-/-*^ L929^*CPSF6*^ cells suppressed the upregulation of these genes (Fig 3H). Furthermore, we also enriched the non-upregulated genes in CPSF6 target genes and found that these genes were mainly enriched to phagocytosis, autophagy, metabolism, protein synthesis and modification, angiogenesis, cell proliferation and apoptosis and other biological processes (S3F and S3G Fig). All these data indicated that the global 3’ UTR shortening caused by CPSF6 deficiency orchestrates the cellular antiviral capacity to inhibit viral infection.

### CPSF6 deficiency increases the use of proximal PASs in many immune-related genes

**Fig 4:**
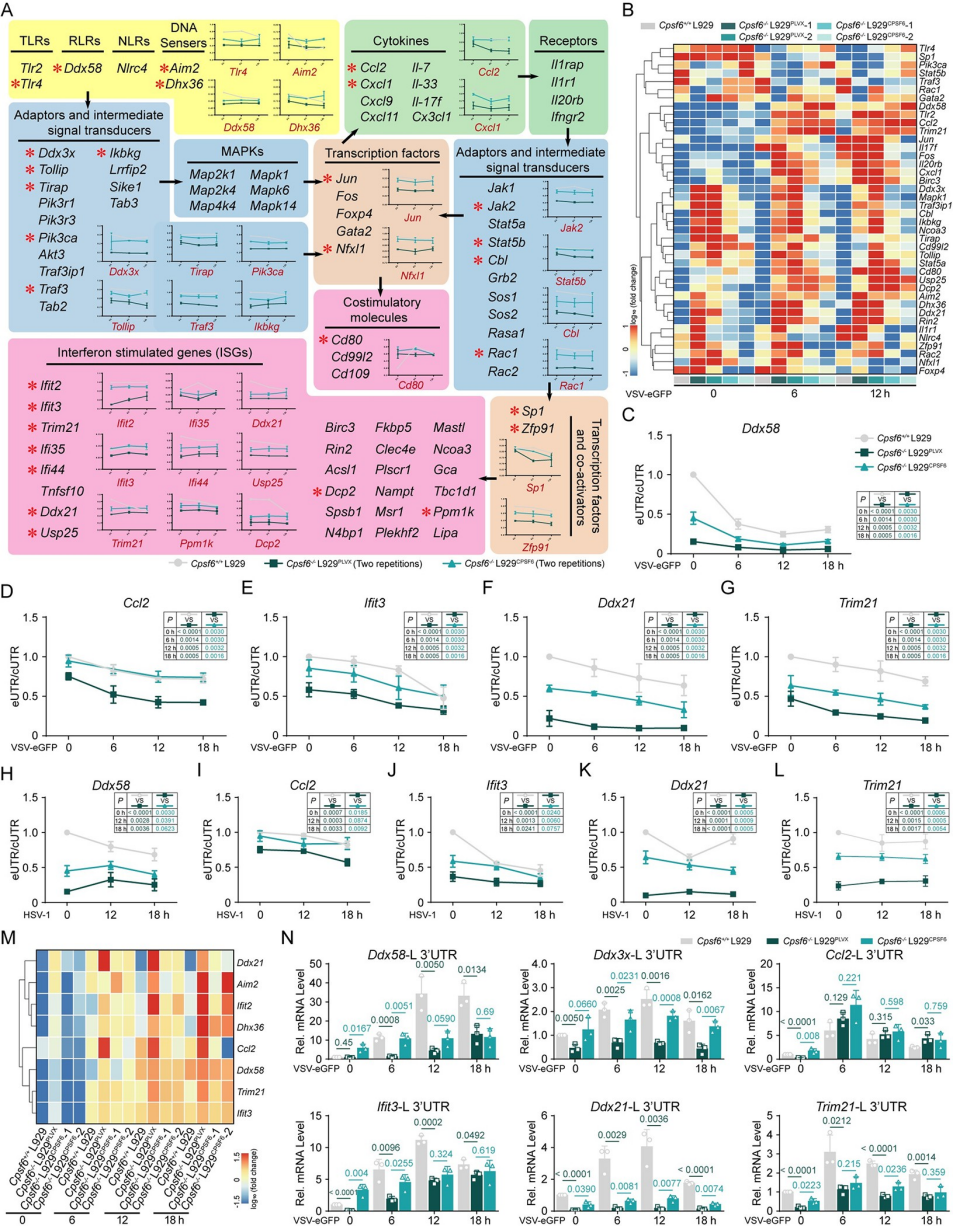
CPSF6 deficiency increases the use of proximal PASs in many immune-related genes. (**a**) The genes with APA involved in virus recognition and signal transduction were shown. The black arrows indicate signal transduction. The x axis of the line graphs denotes infection time points and the y axis denotes normalized 3’ UTR length. Asterisk (*) indicates the APA genes shown in the line graph. (**B**) The heatmap shows mRNA abundance of immune-related genes from (**A**). (**C-G**) qRT–PCR analyses the ratio of longer 3’ UTR isoforms to total mRNA of tested genes *Ddx58* (**C**), *Ccl2* (**D**), *Ifit3* (**E**), *Ddx21* (**F**) and *Trim21* (**G**) in WT, *Cpsf6*^-/-^L929^PLVX^ and *Cpsf6*^-/-^ L929^*CPSF6*^ L929 cells infected with VSV-eGFP at indicated time points. (n = 3 replicates) (**H**-**L**) qRT–PCR analyses the ratio of longer 3’ UTR isoforms to total mRNA of tested genes *Ddx58* (**H**), *Ccl2* (**I**), *Ifit3* (**J**), *Ddx21* (**K**) and *Trim21* (**L**) in WT, *Cpsf6*^-/-^L929^PLVX^ and *Cpsf6*^-/-^ L929^*CPSF6*^ L929 cells infected with HSV-1 at indicated time points. (n = 3 replicates) (**M**, **N**) qRT–PCR analyses the abundance of total mRNA (**M**) or transcripts with long 3’ UTR (**N**) of these tested immune-related genes in WT, *Cpsf6*^-/-^L929^PLVX^ and *Cpsf6*^-/-^ L929^*CPSF6*^ L929 cells infected with VSV-eGFP at indicated time points. (n = 3 replicates). The values represent mean ± SD with individual measurements overlaid as dots, statistical analysis was performed using a two-tailed Student’s *t*-test in (**C**)-(**L**) and (**N**).

More than half of the genes involved in the TLR, RLR or Jak-STAT signalling pathways have multiple PASs in their 3’ UTRs and exhibit noteworthy APA switching to use pPAS after viral infection [29]. Here, our IVT-SAPAS data further confirmed that many immune-related genes involved in virus recognition and signal transduction undergo dynamic APA processing and tend to use pPASs. This preference was even more pronounced in *Cpsf6*^*-/-*^ L929 cells and reversed in *Cpsf6*^*-/-*^ L929^*CPSF6*^ cells (Figs 4A and S4A–S4G). Accordingly, the mRNA abundances of these immune-related genes were significantly increased with the shortening of 3’ UTRs in *CPSF6*^*-/-*^ L929 cells (Fig 4B). To further verify the above data, qRT–PCR analysis was performed to test the APA switching in 5 selected immune-related genes (*Ddx58*, *Ccl2*, *Ifit3*, *Ddx21* and *Trim21*) that are deeply involved in the antiviral immune response. By convention, the segment of the 3’ UTR upstream of the proximal PAS is called the constitutive 3’ UTR (cUTR), while the downstream segment that is present only in longer isoforms is designated the “alternative UTR” (aUTR) or “extended UTR” (eUTR) [2]. Then, we used eUTR/cUTR ratio to indicate the ratio of longer 3’ UTR isoforms to total transcripts. The results were consistent with our IVT-SAPAS data indicating that all tested genes exhibited appreciable 3’ UTR shortening upon VSV-eGFP infection or CPSF6 deficiency, and that ectopic expression of CPSF6 partially prevented this trend (Fig 4C–4G). The same results were obtained in HSV-1-infected L929 cells (Fig 4H–4L) and Poly (I:C)-stimulated L929 cells (S4H–S4K Fig). We next measured the mRNA expression of the tested genes and observed that their expression was markedly increased in *Cpsf6*^*-/-*^ L929 cells and reduced in *Cpsf6*^*-/-*^ L929^*CPSF6*^ cells (Fig 4M). In contrast, the transcripts of these genes with long 3’ UTRs did not show significant upregulation or even downregulation in *Cpsf6*^*-/-*^ L929 cells after VSV infection (Fig 4N). Taken together, these results demonstrated that CPSF6 deficiency leads to the preferential use of pPASs in many immune-related genes.

### The increase in pPAS usage upon CPSF6 deficiency results in increased mRNA stability and protein synthesis

**Fig 5:**
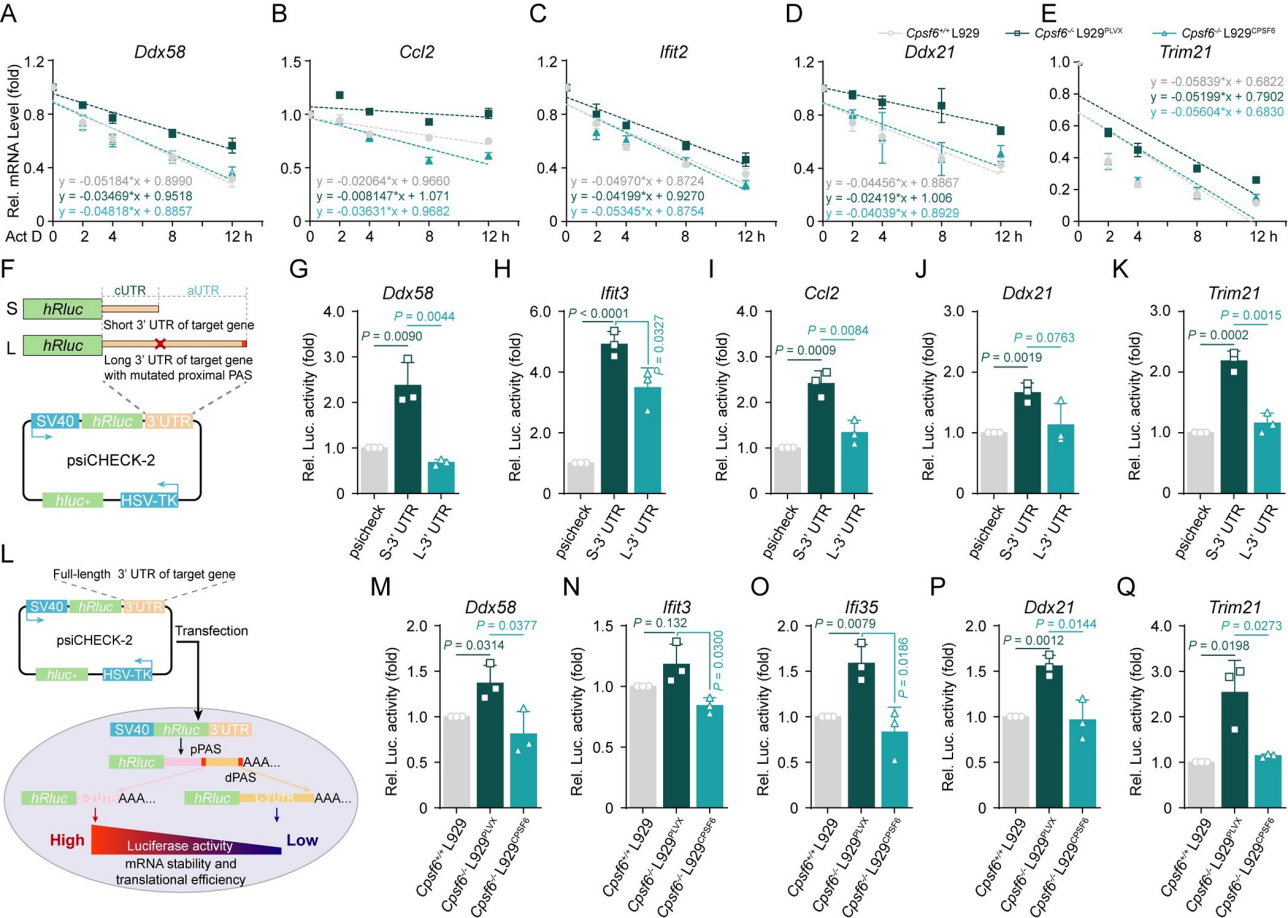
The increase in pPAS usage upon CPSF6 deficiency results in increased mRNA stability and protein synthesis. (**A-E**) qRT–PCR analyses the mRNA abundance of *Ddx58* (**A**), *Ccl2* (**B**), *Ifit2* (**C**), *Ddx21* (**D**) and *Trim21* (**E**) in WT, *Cpsf6*^-/-^L929^PLVX^ and *Cpsf6*^-/-^ L929^*CPSF6*^ L929 cells treated with Act D (5 μg/mL) for the indicated times. (n = 3 replicates) (**F**) Schematic diagram of psiCHECK-3’ UTR recombinant constructs. S, short 3’ UTR; L, long 3’ UTR with mutated proximal PAS. (**G**-**K**) Relative luciferase activities in 293T cells after transfection with reporter vectors bearing S-3’ UTR or L-3’ UTR of *Ddx58* (**G**), *Ifit3* (**H**), *Ccl2* (**I**), *Ddx21* (**J**) and *Trim21* (**K**). *Renilla* luciferase activity was measured and normalized to firefly luciferase activity. (n = 3 replicates) (**L**) Mechanism diagram of PAS selection affecting luciferase protein production. (**M**-**Q**) Relative luciferase activities in WT, *Cpsf6*^-/-^L929^PLVX^ and *Cpsf6*^-/-^ L929^*CPSF6*^ L929 cells after transfection with reporter vectors bearing WT full length 3’ UTR of *Ddx58* (**M**), *Ifit3* (**N**), *Ifi35* (**O**), *Ddx21* (**P**) and *Trim21* (**Q**). (n = 3 replicates). The values represent mean ± SD with individual measurements overlaid as dots, statistical analysis was performed using a two-tailed Student’s *t*-test in (**A**)-(**E**), (**G**)-(**K**) and (**M**)-(**Q**).

Numerous advances in the past few years have substantially enriched our knowledge that 3’ UTRs are hotbeds for mRNA destabilization elements, which often function through the binding of RNA-binding proteins (RBPs) and microRNAs (miRNAs) [43]. Transcripts using pPASs and therefore having shorter 3’ UTRs are generally thought to produce more proteins due to the exclusion of these regulatory elements that mediate degradation or translational efficiency of mRNAs [10]. To test whether the transcripts of these immune-related genes with shortened 3’ UTRs become more stable or more efficiently translated, we first used a miRNA target prediction program (TargetScan) to identify the miRNA binding sites in the long 3’UTR isoform of specific mRNAs. Interestingly, we found that most of the miRNA binding sites in the tested genes, such as *Ddx58*, *Ccl2*, *Ifit2*, *Ddx21*, *Trim21*, *Dhx36* and *Fos*, were localized between the pPAS and the dPAS, a region that specifically presents in the long 3’ UTR (S5A Fig). In agreement with this notion, the extended 3’ UTR region of one tested gene, *Ccl2*, contains conserved seed matches to miR-124a and miR-33 (S5B Fig), both of which have been reported to directly target the degradation of *Ccl2* mRNAs [44,45]. Another tested gene *Fos* has also been demonstrated to be targeted for degradation and translation inhibition by miR-7b and miR-155 that binds to its extended 3’ UTR region in previous studies [46,47] (S5C Fig). These findings raise the intriguing possibility that CPSF6 depletion leading to 3’ UTR shortening may be a mitigation mechanism for miRNA-mediated degradation. Then, we assessed whether CPSF6-mediated APA is implicated in the mRNA stability of targeted genes. When halting transcription with actinomycin D (Act D), we observed that the decay rate of the tested immune-related genes (*Ddx58*, *Ccl2*, *Ifit2*, *Ddx21* and *Trim21*) was slower in *Cpsf6*^*-/-*^ L929 cells, but faster in *Cpsf6*^*-/-*^ L929^*CPSF6*^ cells (Fig 5A–5E). The above results indicated that the increased usage of pPASs in CPSF6-deficient L929 cells resulted in an increased mRNA stability of those immune-related genes.

To further elucidate the effect of CPSF6-mediated APA switch on the ultimate protein translation from the immune-related genes, 5 target genes (*Ddx58*, *Ifit3*, *Ccl2*, *Ddx21* and *Trim21*) were selected to perform luciferase reporter gene assays. The short 3’ UTRs formed by using the pPAS of each target genes were inserted downstream of the *Renilla* luciferase translational stop codon in the psiCHECK-2 plasmid, and the resulting plasmid was named the S-3’ UTR construct. Similarly, L-3’ UTR construct contained the long 3’ UTR of the target gene with both the proximal and distal PASs, but the pPAS was mutated to prevent the formation of the S-3’ UTR isoform (Fig 5F). We observed that the expression of short 3’ UTRs of these tested genes increased *Renilla* luciferase expression (Fig 5G–5K), implying the increased translation efficiency of these target genes with short 3’ UTRs. Finally, we used reporter assays to measure the luciferase activity of reporter plasmids containing the *Ddx58*, *Ifit3*, *Ifi35*, *Ddx21* or *Trim21* wild-type 3’ UTR in different lines of L929 cells (Fig 5L). The results showed that the luciferase activity of these reporters was increased in *Cpsf6*^*-/-*^ L929 cells but decreased in *Cpsf6*^*-/-*^ L929^*CPSF6*^ cells (Fig 5M–5Q). Together, our data suggested that the increased abundance of proteins translated from immune-related genes upon CPSF6 depletion is mediated by shortening of their 3’ UTRs, which may promote activation of the antiviral immune response.

### CPSF6 deficiency promotes IFN-I signalling activation

**Fig 6:**
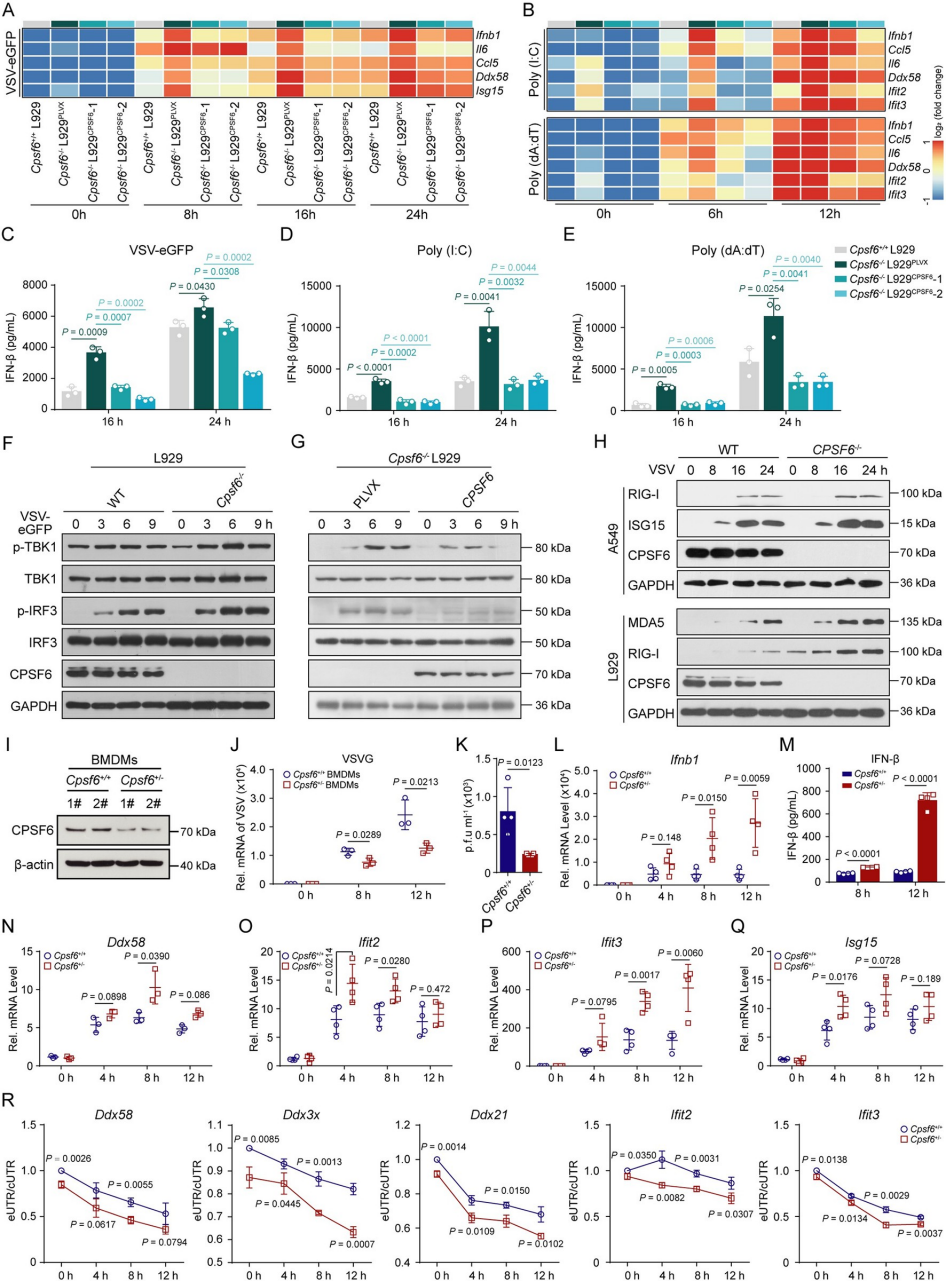
CPSF6 deficiency promotes IFN-I signalling activation. (**A**, **B**) The heatmap shows the mRNA abundance of *IFNB* and ISGs in WT, *Cpsf6*^-/-^L929^PLVX^ and *Cpsf6*^-/-^ L929^*CPSF6*^ L929 cells infected with VSV-eGFP (**A**) or stimulated with poly (I:C) or Poly (dA:dT) (**B**) at indicated time points. (n = 3 replicates) (**C-E**) ELISA analyses of IFN-β secretion in cell supernatant from WT, *Cpsf6*^-/-^L929^PLVX^ and *Cpsf6*^-/-^ L929^*CPSF6*^ L929 cells infected with VSV-eGFP (**C**) or stimulated with poly (I:C) (**D**) or Poly (dA:dT) (**E**) at indicated time points. (n = 3 replicates) (**F**, **G**) Immunoblot analyses of phosphorylated (p-) TBK1 and IRF3 in WT, *Cpsf6*^-/-^L929^PLVX^ and *Cpsf6*^-/-^ L929^*CPSF6*^ L929 cells infected with VSV-eGFP at indicated time points. (**H**) Immunoblot analyses of the protein expression of ISGs in WT and *CPSF6*^-/-^ A549 and L929 cells infected with VSV-eGFP at indicated time points. (**I**) Immunoblot analyses knocking-down efficiency of CPSF6 in BMDMs from *Cpsf6*^*+/+*^ and *Cpsf6*^*+/-*^ mice. (**J**) qRT–PCR analyses of VSV mRNA abundance in *Cpsf6*^*+/+*^ and *Cpsf6*^*+/-*^ BMDMs infected with VSV-eGFP at indicated time points. (n = 3 replicates) (**K**) Plaque assays of VSV titers in cell supernatants from *Cpsf6*^*+/+*^ and *Cpsf6*^*+/-*^ BMDMs infected with VSV-eGFP for 12 h. (n = 4 replicates) (**L**) qRT–PCR analyses of *Ifnb1* mRNA abundance in *Cpsf6*^*+/+*^ and *Cpsf6*^*+/-*^ BMDMs infected with VSV-eGFP at indicated time points. (n = 4 replicates) (**M**) ELISA analyses of IFN-β secretion in cell supernatant from *Cpsf6*^*+/+*^ and *Cpsf6*^*+/-*^ BMDMs infected with VSV-eGFP at indicated time points. (n = 4 replicates) (**N**-**Q**) qRT–PCR analyses of *Ddx58* (**N**), *Ifit2* (**O**), *Ifit3* (**P**) and *Isg15* (**Q**) mRNA abundance in *Cpsf6*^*+/+*^ and *Cpsf6*^*+/-*^ BMDMs infected with VSV-eGFP at indicated time points. (n = 4 replicates) **R**, qRT–PCR analyses the ratio of longer 3’ UTR isoforms to total mRNA of tested genes *Ddx58*, *Ddx3x*, *Ddx21*, *Ifit2* and *Ifit3* mRNA abundance in *Cpsf6*^*+/+*^ and *Cpsf6*^*+/-*^ BMDMs infected with VSV-eGFP at indicated time points. (n = 3 replicates). Data are representative of three independent experiments, with one representative shown in (**F**)-(**I**). The values represent mean ± SD with individual measurements overlaid as dots, statistical analysis was performed using a two-tailed Student’s *t*-test in (**C**)-(**E**) and (**J**)-(**Q**).

To further determine the effects of CPSF6 deficiency on virus replication, we evaluated the virus-induced activation of IFN-I signalling in *CPSF6*^*-/-*^ A549 and L929 cells. As expected, the mRNA expression of *IFNB*, *IL6*, and a number of ISGs (*CCL5*, *DDX58*, *IFIT1* and *ISG15*) was obviously increased in *CPSF6*^*-/-*^ A549 cells infected with VSV-eGFP or SeV compared with the corresponding wild-type cells (S6A Fig). After infection with VSV-eGFP, or treatment with Poly (I:C) or Poly (dA:dT), *Cpsf6*^*-/-*^ L929 cells also exhibited higher expression of *IFNB* and ISGs, but this increase was suppressed in *Cpsf6*^*-/-*^ L929^*CPSF6*^ cells (Fig 6A and 6B). Furthermore, higher levels of secreted IFN-β were detected in culture supernatant from *CPSF6*^*-/-*^ A549 and L929 cells (Figs 6C–6E and S6B–S6D), and this increasing trend was abolished by ectopic expression of CPSF6 (Fig 6C–6E), demonstrating that CPSF6 deficiency promoted IFN-I responses against viral infection by increasing IFN-β production and ISG expression. Upon viral infection, germline-encoded PRRs are employed to detect viral nucleic acids and then trigger the TBK1-IRF3 signalling cascade to produce IFN-Is and proinflammatory cytokines [21]. To further unveil the function of CPSF6 in virus-triggered innate immune responses, we measured the levels of phosphorylated IRF3 and TBK1 and observed that CPSF6 deficiency markedly facilitated TBK1 and IRF3 phosphorylation after VSV-eGFP infection (S6E Fig). The same results were also found in VSV-eGFP *CPSF6*^*-/-*^ L929 cells (Fig 6F), while *Cpsf6*^*-/-*^ L929^*CPSF6*^ cells with ectopic expression of CPSF6 showed the opposite pattern (Fig 6G). With the marked increase in IRF3 phosphorylation, the expression of ISG-encoded proteins, such as MDA5, RIG-I and ISG15, also increased significantly in *CPSF6*^*-/-*^ A549 and L929 cells compared with wild-type cells (Figs 6H, S6F and S6G). Accordingly, ectopic expression of CPSF6 in *Cpsf6*^*-/-*^ L929^*CPSF6*^ cells inhibited the expression of ISGs upon VSV-eGFP infection and Poly (I:C) or Poly (dA:dT) stimulation (S6H and S6I Fig).

In addition, we also tried to study the *in vivo* roles of CPSF6 in mice by using CRISPR/Cas9 genome editing to delete the third exon of *Cpsf6*. However, after zygote microinjection, we only obtained 12 infertile *Cpsf6*^*+/-*^ mice, of which 2 males were dissected and found to have clear epididymides without sperm. After evaluating the expression of CPSF6, we found that it was significantly lower in BMDMs from *Cpsf6*^*+/-*^ mice than in macrophages from their wild-type littermates (Fig 6I). Then, a lower viral load and greater IFN-β production were observed in BMDMs from *Cpsf6*^*+/-*^ mice (Fig 6J–6M). Accordingly, the mRNA expression of many ISGs, such as *Ddx58*, *Isg15*, *Ifit2* and *Ifit3*, was also higher in *Cpsf6*^*+/-*^ BMDMs (Fig 6N–6Q). Likewise, the 3’ UTRs of *Ddx58*, *Ddx3x*, *Ddx21*, *Ifit2* and *Ifit3*, were significantly shorter in primary BMDMs from *Cpsf6*^*+/-*^ mice, and such shortening was more pronounced upon VSV-eGFP infection (Fig 6R). These data convincingly demonstrated that the downregulation of CPSF6 promoted interferon production by promoting the use of proximal PASs in immune-related genes, which may amplify IFN-I-dependent antiviral immune responses against both RNA and DNA viruses.

## Discussion

**Fig 7:**
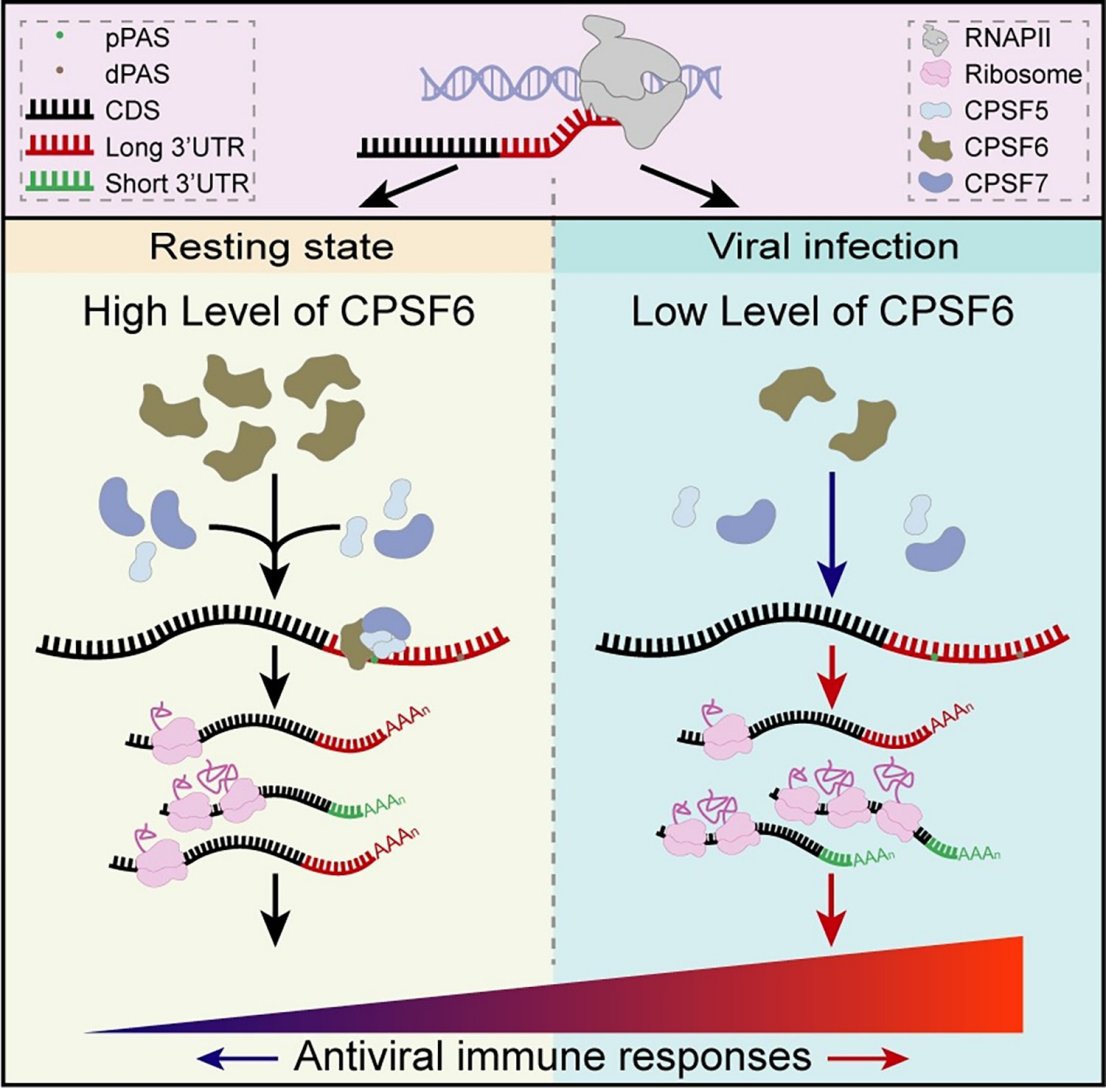
The mechanism by which CPSF6 regulates antiviral immune responses through APA. CPSF6 is a negative regulator of IFN-I signalling in resting cells, and the protein abundance of CPSF6 decreases upon viral infection. With the decrease in CPSF6 expression, proximal PASs are preferentially selected in immune-related genes, resulting in the production of transcripts with short 3’ UTRs. The translational output of immune-related transcripts with shortened 3’ UTRs increases due to the increased mRNA stability and translation efficiency, which ultimately increases the intensity of activated IFN-I signalling-dependent antiviral immune responses.

APA occurring on the 3’ UTRs is an important posttranscriptional regulatory mechanism and is involved in many physiological and pathological processes, particularly in cancer cells and activated immune cells, as well as in antibacterial and antiviral immune responses [29,48–50]. We previously described a dynamic landscape of tandem 3’ UTRs during viral infection [29]. Here, we further identified that the protein expression of CPSF6 was significantly and universally reduced facing multiple viral infection, leading to the global usage of pPASs and the preferential generation of transcripts with short 3’ UTRs. Since the transcripts of many immune-related genes with shortened 3’UTRs had improved mRNA stability and translation efficiency, downregulation of CPSF6 during viral infection promotes the protein output of these transcripts, thereby accelerating the activation of the IFN-I signalling response and the rapid clearance of invading viruses (Fig 7). As a pivotal cellular host factor, CPSF6 also protects the viral genome or proteins to allow them to translocate from the cell periphery into the nucleus in an APA-independent manner. For example, CPSF6 interacts with the HIV-1 capsid protein to promote the nuclear import of the preintegration complex (PIC) and its targeting to active chromatin for integration [51]. Moreover, HIV-1 capsid mutants with an impaired interaction with CPSF6 cannot replicate in human macrophages due to the establishment of IFN-I-based antiviral state [52]. CPSF6 also interacts with the NP1 protein of human bocavirus 1 (HBoV1) and minute virus of canines (MVC) and mediates the nuclear import of NP1 to regulate the maturation of viral mRNAs encoding capsid proteins as well as the replication of viral DNA [53,54]. All these studies revealed that CPSF6 not only affects viral replication directly but also regulates the host antiviral immune responses, suggesting the importance of CPSF6 as a target in the development of antiviral therapies.

APA usually occurs by activation of ‘weaker’ PASs located upstream of ‘stronger’ PASs. The activation of the ‘weaker’ PASs is mainly controlled and determined by the abundance or activity of core processing factors [55]. In this study, we found that the expression of CPSF6 was significantly decreased after viral infection as well as bacterial and helminthic infection (Fig 1). Furthermore, we analyzed some datasets related to immune diseases (GSE46922/16032/13887/93776/166388/4479/27628), and a similar trend in CPSF6 expression was found in patients with paediatric immune thrombocytopenia (ITP), severe asthma, rheumatoid arthritis (RA), systemic lupus erythematosus (SLE) or psoriasis (S7A–S7E Fig), and in mice with sepsis induced by caecal ligation and puncture (CLP) (S7F Fig) or psoriasis induced by imiquimod (IMQ) (S7G Fig). The CFIm complex preferentially binds UGUA motifs [56], which are typically located 40–50 nt upstream of the distal cleavage and polyadenylation site [57]. Reduced availability of CFIm leads to decreased use of UGUA-containing distal PASs, whereas proximal sites lacking the UGUA motif are not affected by CFIm loss [43]. Our study confirmed that decreased expression of CPSF6, a CFIm subunit, can significantly promote the use of proximal PASs in a large number of genes. Moreover, CPSF6-mediated APA regulation plays a broad and important role in the maintenance or imbalance of immune homeostasis. Therefore, it is of great importance to clarify the regulation of CPSF6 expression to elucidate the physiological function of CPSF6-mediated APA. Previous studies have shown that the CPSF6 activity can be regulated by phase separation [58] and multiple posttranslational modifications, which are thought to be essential for proper functioning and regulation of 3’ end processing [43]. For example, Ser166 in the RNA recognition motif (RRM) of CPSF6 is subject to phosphorylation [59], while arginine in the glycine-arginine rich (GAR) motif can be methylated *in vitro* by the methyltransferase PRMT5 [60]. The arginine/serine (RS)-like domain (RSLD) also has different phosphorylation states [61]. Furthermore, CPSF6 undergoes liquid-liquid phase separation to recruit CPSF5 into foci, which is associated with APA regulation [58]. Here, using the *miRSystem* prediction programs, we further identified several potential binding sites for miRNAs in the 3’ UTR of *CPSF6* mRNA (S7H Fig), particularly 7 complementary binding sites (seedregion) for miR-377-3p with high evolutionary conservation (S7I and S7J Fig). miR-377-3p has been shown to play a role in the regulation of inflammation [62,63], and its level is increased in PBMCs and serum from SLE patients [64]. Upregulation of miR-377-3p was also observed upon viral infection (S7K Fig). Through overexpressing mimic miR-377 (S7L Fig), we found the expression of CPSF6 was down-regulated (S7M and S7N Fig). Accordingly, mRNA decay assays showed that *Cpsf6* mRNAs became more unstable after VSV-eGFP infection (S7O Fig) or Poly (I:C) stimulation (S7P Fig), implicating that the mRNA stability of CPSF6 may be affected by miR-377-3p. Thus, the expression of CPSF6 upon viral infection may be regulated by multidimensional mechanisms, further highlighting the functional importance and adjustability of CPSF6.

In addition to regulating the antiviral immune responses and orchestrating virus integration, CPSF6 is also involved in cancer progression and immunosuppression. Different studies have reported that the high expression of CPSF6 may be a novel diagnostic and prognostic marker for several tumours, such as hepatocellular carcinoma (HCC) [65], esophageal squamous cell carcinoma (ESCC) [66], lung adenocarcinoma (LUAD) [67] and breast cancer [68]. We also analyzed The Cancer Genome Atlas (TCGA) data from the TIMER database and found that CPSF6 is significantly high expressed in multiple tumour tissues than in normal tissues (S8A Fig). Moreover, patients with a high mRNA level of CPSF6 had a worse prognosis and shorter overall or disease-free survival time than those with a low CPSF6 level (S8B–S8F Fig). Thus, in addition to its regulatory role in the proliferation, tumorigenicity, and cell death of tumour cells [67–69], the role of immunosuppression induced by high expression of CPSF6 in tumour progression should also attract more attentions. Interestingly, the expression of miR-377, which often functions as a tumour suppressor [70], is downregulated in multiple cancer types (S8G Fig), further suggesting a significant negative correlation between miR-377 and CPSF6 expression. Overall, we demonstrate that the expression dynamics of CPSF6 and its effect on APA processing can fine-tune the host immune responses, a finding that may have implications for developing new therapeutic strategies against pathogen infections or cancers.

## Materials and methods

### Ethics statement

All animal experiments were performed in accordance with the Guidelines for the Care and Use of Laboratory Animals, with the approval of the Institutional Animal Care and Use Committee (IACUC) of Sun Yat-sen University (Guangzhou, China).

### Cell culture

A549, L929 and 293T cells were purchased from ATCC. All these cells were cultured in endotoxin-free DMEM (Gibco), supplemented with 10% FBS (Gibco) and 1% penicillin–streptomycin (Gibco). All cells were incubated at 37°C with 5% CO_2_.

Mouse bone marrow derived macrophages (BMDMs) were isolated from the bone marrow of 6–8 weeks old female C57BL/6J mice (Guangdong Medical Laboratory Animal Center) and cultured for 6–8 days with 50 ng/mL macrophage colony-stimulating factor (PeproTech).

### Mice

*Cpsf6*^+/+^ and *Cpsf6*^+/−^ mice (6–8 weeks old) were generated by Biocytogen Pharmaceuticals (Beijing) Co., Ltd using the CRISPR/Cas9 approach. Mice were housed and bred under specific pathogen-free (SPF) conditions.

### Antibodies and reagents

Anti-CPSF5 (1:5000; 10322-1-AP, Proteintech), anti-CPSF6 (1:5000; ab175237, Abcam), anti-CPSF7 (1:2000; 55195-1-AP, Proteintech), anti-RIG-I (1:1000; D14G6, Cell Signaling), anti-MDA5 (1:1000; D74E4, Cell Signaling), anti-IFIT3 (1:2000; 15201-1-AP, Proteintech), anti-ISG15 (1:2000; 15981-1-AP, Proteintech), anti-DDX21 (1:2000; 10528-1-AP, Proteintech), anti-DHX15 (1:2000; 12265-1-AP, Proteintech), anti-CCL2 (1:2000; 66272-1-Ig, Proteintech), anti-IRF3 (1:1,000; sc-33641, Santa Cruz), anti-phosphorylated IRF3 (1:1000; 4D4G, Cell Signaling), anti-TBK1 (1:1000; D1B4, Cell Signaling), anti-phosphorylated TBK1 (1:1000; D52C2, Cell Signaling), anti-VSVG (1:1000; V5507, Sigma), anti-ICP27 (1:1000; ab31631, Abcam), anti-GAPDH (1:20000; 60004-1-Ig, Proteintech), anti-HA (1:5000; clone HA-7, H6533, Sigma), anti-Flag (1:5000; clone M2, F1804, Sigma), Anti-rabbit IgG (H+L), (Alexa Fluor 555 Conjugate) (1:1000, 4413, Cell Signaling), goat anti-mouse IgG-HRP (1:10000; HA1006, HuaBio), goat anti-rabbit IgG-HRP (1:10000; HA1001, HuaBio). The poly(I:C) (LMW) and poly(dA:dT) were purchased from InvivoGen. Lipofectamine 3000 and Lipofectamine RNAiMAX were purchased from Invitrogen. Act D (actinomycin D) was purchased from Sigma.

### Generation of *CPSF6*^−/−^ cells

*CPSF6*^−/−^ A549 and *Cpsf6*^−/−^ L929 cells were constructed using the CRISPR/Cas9 genome editing technology. Small guide RNAs (sgRNAs) targeting the genomic sequences of target genes were cloned and inserted into the PX458 vector. These CRISPR plasmids were transfected into A549 or L929 cells using Lipo3000, and single-cell colonies of GFP^+^ cells were picked and validated at the translational level by western blot analysis. The sgRNA targeting sequences were as follows:

sgRNA-1: 5’-CACCGTCCATGTAATCTCGGTCTTC-3’ for A549 cells,

sgRNA-2: 5’-CACCGCGGGCAAATGGCCAGTCAAA-3’ for A549 cells;

sgRNA-1: 5’-CACCGATGCCAACATCAGATAGTCG-3’ for L929 cells,

sgRNA-2: 5’-CACCGATGACGGTATTCTCGCTCT-3’ for L929 cells.

### Generation of CPSF6-reconstituted L929 cells (*Cpsf6*^*-/-*^ L929^*CPSF6*^ cells)

The CDS of CPSF6 was subcloned and inserted into the pCDH-CMV-MCS-EF1-Puro plasmid. This vector was transfected into HEK293T cells along with the following two lentiviral packing plasmids: psPAX2 and pMD2.G. Culture supernatants were then collected 48 hr after transfection. The culture supernatant was concentrated by ultracentrifugation before use for infection. Infection-positive *CPSF6*^−/−^ L929 cells were selected and enriched on the basis of resistance to puromycin. Monoclonal cells were screened by a limiting dilution assay and confirmed by sequencing of PCR- amplified fragments. Immunoblot analysis of cell lysates was performed with the corresponding antibody.

### Analogue stimulation and virus infection

Cells were stimulated with HSV60 (1 μg ml^−1^), Poly (I:C) (1 μg ml^−1^) or Poly (dA:dT) (1 μg ml^−1^) delivered via polyethylenimine (PEI) for the indicated hours. Cells were infected with viruses at the indicated MOI in serum-free DMEM for 1 hr, washed with 1×PBS, and incubated with fresh complete medium for the times shown in the figures.

### Plaque assay

Culture the Vero cells in growth medium (DMEM+10% FBS) in a six-well plate to give 90–100% confluence by the following days. Then, dilute the virus suspension tenfold serially from 1:10^−1^ to 1:10^−6^, and infect the confluent monolayers of Vero cells with 400 μL volume of diluted virus and 600 μL volume of serum-free DMEM (total 1000 μL volume/well). After incubation 1 hr, discard the unadsorbed virus and overlay the monolayer with 3 mL of autoclaved agar overlay (at about 40°C). After the gel solidifies, incubate the plates at 37°C and 5% CO_2_ for up to 24–48 hr. After 24–48 hrs, stain the plates with 0.1% neutral red in PBS for 1 hr (1 mL/well) followed by counting the plaques. Calculate the virus titer using the following formulas:

Pfu/mL = Average no. of plaques per dilution/(D*V)

*D*: Dilution factor (for dilution 10^−2^; D is 0.01 and for dilution 10^−4^; D is 0.0001).

*V*: Volume of virus added per well (in mL; 0.4 mL in the above protocol).

### Preparation of the IVT-SAPAS library, APA sequencing and analysis

The IVT-SAPAS libraries were constructed based on previously developed SAPAS methods [38,39]. Briefly, 200 ng of total RNA was randomly fragmented by heating, and the first round of reverse transcription was performed using an anchored oligo d(T) primer containing an Illumina A adaptor and the T7 promoter sequence. The second strand was synthesized with RNase H, *Escherichia coli* DNA polymerase and DNA ligase. Subsequently, the RiboMAX Large Scale RNA Production System-T7 (Promega) was used to perform *in vitro* transcription according to the manufacturer’s instructions (Promega). RNA products were purified with the Agencourt RNA Clean XP kit (Beckman Coulter). A second round of reverse transcription was conducted using random primers containing a partial Illumina B adaptor sequence. PCR was then performed to amplify the cDNA, 200–500 bp fragments were purified with AMPure XP Beads (Beckman Coulter), and the quality of the library was evaluated with an Agilent 2100 Bioanalyzer. The final pooled libraries were quantified and sequenced with an Illumina HiSeq 2500 Sequencing System.

Raw reads were first trimmed, filtered and mapped to the mouse genome (mm9 downloaded from the UCSC Genome Bioinformatics website). The unique mapped reads were used to filter for internal priming by examining the genomic sequences 1 to 20 bases downstream of the poly(A) cleavage sites. To eliminate bias introduced by unequal read counts at different time points, the total counts at each time point were normalized to one million. Poly(A) site definition and tandem 3’ UTR annotation were performed as previously described. The isoform-weighted 3’ UTR length was defined as the mean 3’ UTR length weighted by the read counts for each tandem 3’ UTR isoform, and the normalized 3’ UTR length was defined as the percentage of the isoform-weighted 3’ UTR length relative to the longest tandem 3’ UTR length across all time points. We used a linear model to assess APA switching events, and genes with an absolute tandem 3’ UTR switching index (TSI) value of 40.1 and a *P* value of less than the threshold corresponding to a BH-sense FDR of 0.01 was defined as an APA gene.

### Luciferase reporter assay

The 3’ UTRs of mouse target genes were amplified from the L929 cell genome by a proofreading Pfu polymerase (Takara) and sequenced to confirm their authenticity. Mutation of proximal poly(A) sites was performed using a Site-directed Gene Mutagenesis Kit (ExCell Bio), and 3’ UTRs of differing lengths were cloned and inserted into the psiCHECK-2 plasmid. For reporter assays, 293T cells were plated at a density of 10^5^ cells per well in a 48-well plate. The cells in each well were transiently transfected for 24 hr with psiCHECK-2 plasmids with different 3’ UTRs inserted downstream of the *Renilla* luciferase gene using ViaFect Transfection Reagent (Promega). Subsequently, the cells were lysed and collected for luciferase reporter assays. The luciferase activity in cell lysates was measured with a dual luciferase reporter gene assay system (Promega) according to the manufacturer’s instructions. Firefly luciferase reporters in psiCHECK-2 plasmids were used as internal controls. Each experiment was performed in triplicate and repeated three times. The data are presented as the mean ± s.d. values.

### Quantitative real-time PCR (qRT–PCR) analysis

For mRNA analysis, total RNA was extracted using TRIzol reagent (Invitrogen) and reverse transcribed with a PrimeScript RT Reagent Kit with gDNA Eraser (TaKaRa) according to the manufacturer’s instructions. A Light Cycler 480 instrument (using the 384-well module) with 2×Polarsignal qPCR mix (MIKX) was used for quantitative real-time PCR analysis. Target mRNA expression levels were normalized to the expression level of β-actin in each individual sample. The 2^−ΔΔCt^ method was used to calculate relative expression changes. For microRNA analysis, 1 **μ**g of total RNA was reverse transcribed with a miRNA First Strand cDNA Synthesis kit (Tailing Reaction) (Sangon Biotech) according to the manufacturer’s instructions. A Light Cycler 480 instrument (using the 384-well module) with 2×Polarsignal qPCR mix (MIKX) was used for quantitative real-time PCR analysis. U6 was used as the control for miRNA expression, and the relative expression of microRNAs was calculated by the 2^-ΔΔCt^ method. S1 Table lists the primers detail.

### RNA interference

LipoRNAiMAX (Invitrogen) was used for transfection of siRNAs (30 nM) into A549 or L929 cells according to the manufacturer’s instructions. The sequences of the siRNAs are as follows:

Human-CPSF6-siRNA: GGATCAAGACGTGAACGAT

Mouse-CPSF6-siRNA: CGACTACATGGATACTCTT

### RNA decay assay

L929 cells were seeded at a density of 4 × 10^5^ cells/mL in 12-well plates. After incubation overnight, the cells were treated with the transcription inhibitor Act D (5 μg/mL, Sigma) to block *de novo* RNA synthesis, and collected at the indicated times. RNA samples were extracted for qRT–PCR to measure the mRNA levels of the indicated genes.

### Immunoblotting

Cells were lysed with cell lysis buffer (Cell Signaling Technology) supplemented with protease inhibitor ‘cocktail’ (Roche). Protein concentrations in the extracts were measured by a BCA assay (Pierce). A total of 40 mg of protein in each sample was separated by sodium dodecyl sulfate–polyacrylamide gel electrophoresis (SDS–PAGE) and then electrotransferred to a polyvinylidene difluoride membrane (Hybond-P; GE Healthcare Life Sciences). Membranes were incubated with the indicated primary antibodies followed by HRP-conjugated secondary antibodies. Bands were detected with an Immobilon ECL kit (Millipore) and imaged on X-ray films (Kodak, Xiamen, China).

### Flow cytometry

A549 and L929 cells were transiently transfected with siRNAs for 48 hr and then challenged with VSV-eGFP for 6 hr at an MOI of 2. The cells were washed once in cold PBS and resuspended in cold staining buffer (PBS with 1% fetal bovine serum). For each condition, 10,000 cells were counted, and subsequent analyses were performed on a flow cytometry system.

### Statistics and reproducibility

Statistical analyses were carried out using Microsoft Excel software and GraphPad Prism to assess the differences between experimental groups. Statistical significance was determined using a two-tailed, unpaired Student’s *t* test or the Mann-Whitney U test with a confidence interval of 95%. P ≤ 0.05 was considered to indicate a statistically significant difference. All experiments were performed three or more times independently under identical or similar conditions.

## Supporting information

S1 Fig CPSF6 protein expression is decreased in virus-infected cells. (**A**, **B**) Immunoblot analyses the CFIm complex expression in BMDMs infected with VSV-eGFP or HSV-1 (**A**) or stimulated with poly (I:C) or Poly (dA:dT) (**B**) at indicated time points. Data are representative of three independent experiments, with one representative shown in (**A** and **B**).
(TIF)

S2 Fig CPSF6 deficiency inhibits virus replication. (**A**) qRT–PCR analyses of the knockdown efficiency of CPSF6 in A549 and L929 cells. (**B**) FACS analyses of VSV replication in CPSF6 knocked down A549 or L929 cells infected with VSV-eGFP for 12h. (**C**) qRT–PCR analyses of VSV replication in CPSF6 knocked down A549 and L929 cells infected with VSV-eGFP at indicated time points. (**D**) Fluorescence microscope analyses of GFP intensity in WT and *CPSF6*^-/-^ A549 cells infected with VSV-eGFP for 16 or 24 h. Scale bar, 100 μm. (**E**, **F**) qRT–PCR analyses of SeV (**E**) or HSV-1 (**F**) replication in WT and *Cpsf6*^-/-^ L929 cells. (**G**) Immunoblot analyses of the ICP27 expression in WT and *Cpsf6*^-/-^ L929 cells infected with HSV-1 at indicated time points. (**H**) qRT–PCR analyses of HSV-1 replication in *Cpsf6*^-/-^L929^PLVX^ and *Cpsf6*^-/-^ L929^*CPSF6*^ L929 cells. (**I**) Immunoblot analyses of the ICP27 expression in *Cpsf6*^-/-^L929^PLVX^ and *Cpsf6*^-/-^ L929^*CPSF6*^ L929 cells infected with HSV-1 at indicated time points. Data are representative of three independent experiments, with one representative shown in (**D**), (**G**) and (**I**). The values represent mean ± SD with individual measurements overlaid as dots, statistical analysis was performed using a two-tailed Student’s *t*-test in (**A**-**C**), (**E**), (**F**) and (**H**).
(TIF)

S3 Fig CPSF6 deficiency leads to global 3’ UTR shortening. (**A**) Genomic locations of reads uniquely mapped to the nuclear genome after internal priming filtering. (**B**) Distribution of classical and non-classical poly(A) signal hexamer at different genomic locations. (**C**) Number of genes with different numbers of poly(A) sites. (**D**, **E**) The number of poly (A) site-switched genes in L929 cells infected with VSV-eGFP for 6 (**D**) or 12 h (**E**). (**F**, **G**) KEGG enrichment (**F**) and GO (**G**) analysis of the non-immune-related genes among the CPSF6 target genes.
(TIF)

S4 Fig CPSF6 deficiency increases the use of proximal PASs in many immune-related genes. (**A-G**) IVT-SAPAS read alignments of *Cxcl1* (**A**), *Ccl2* (**B**), *Rac1* (**C**), *Il20rb* (**D**), *Ddx3x* (**E**), *Dhx36* (**F**) and *Traf3* (**G**) are shown. (**H-K**) qRT–PCR analyses the ratio of longer 3’ UTR isoforms to total mRNA of tested genes *Ddx58* (**H**), *Ccl2* (**I**), *Ifit2* (**J**) and *Ifit3* (**K**) in WT, *Cpsf6*^-/-^L929^PLVX^ and *Cpsf6*^-/-^ L929^*CPSF6*^ L929 cells stimulated with poly (I:C) at indicated time points. (n = 3 replicates). The values represent mean ± SD with individual measurements overlaid as dots, statistical analysis was performed using a two-tailed Student’s *t*-test in (**H**-**K**).
(TIF)

S5 Fig Expression of CPSF6 target genes may be regulated by miRNAs. (**A**) The number of miRNAs binding to immune-related gene 3’ UTRs was predicted by a miRNA target prediction program (TargetScan). (**B** and **C**) Binding diagram of specific microRNAs in the 3’ UTR region of *Ccl2* (**B**) and *Fos* (**C**) transcripts.
(TIF)

S6 Fig CPSF6 deficiency promotes IFN-I signalling activation. (**A**) The heatmap shows the mRNA abundance of *IFNB* and ISGs in WT and *CPSF6*^-/-^ A549 cells infected with VSV-eGFP or SeV at indicated time points. (**B-D**) ELISA analyses the secretion of INF-β in cell supernatant from WT and *CPSF6*^-/-^ A549 cells infected with VSV-eGFP (**B**) or SeV (**C**) or stimulated with Poly (dA:dT) (**D**) at indicated time points. (**E**) Immunoblot analyses of phosphorylated (p-) TBK1 and IRF3 in WT and *CPSF6*^-/-^ A549 cells infected with VSV-eGFP at indicated time points. (**F**) Immunoblot analyses of the protein expression of ISGs in WT and *CPSF6*^-/-^ A549 cells infected with SeV at indicated time points. (**G**) Immunoblot analyses of the protein expression of ISGs in WT and *Cpsf6*^-/-^ L929 cells stimulated with Poly (I:C) at indicated time points. (**H**, **I**) Immunoblot analyses of the protein expression of ISGs in *Cpsf6*^-/-^ L929^PLVX^ and *Cpsf6*^-/-^ L929^*CPSF6*^ L929 cells infected with VSV-eGFP (**H**) or stimulated with Poly (I:C) (**I**) at indicated time points. Data are representative of three independent experiments, with one representative shown in (**E**-**I**). The values represent mean ± SD with individual measurements overlaid as dots, statistical analysis was performed using a two-tailed Student’s *t*-test in (**B**-**D**).
(TIF)

S7 Fig The mRNA expression of CPSF6 is regulated by virus-induced miR-377-3p. (**A-G**) The relative mRNA expression of *CPSF6* in peripheral blood-T cells from ITP (**A**), PBMCs from asthma (**B**), monocytes from patients with RA (**C**), CD3^+^ T cells from patients with SLE (**D**), epidermis from psoriasis (**E**), CD4^+^ splenocytes from CLP-induced sepsis (**F**) and skin from IMQ-induced psoriasis (**G**) are shown. (**H**) The prediction of potential binding miRNAs on *CPSF6* mRNA using the *miRSystem* database. (**I**) The schematic diagram of the binding of miR-377-3p and CPSF6-3’UTR. (**J**) Multiple sequence alignment of the *CPSF6*-3’ UTR showed the conservation of seedregion in tetrapods. (**K**) qRT–PCR analyses the expression of predicted miRNAs in L929 cells infected with VSV-eGFP or HSV-1 for the indicated times. (**L** and **M**) qRT–PCR analyses the expression of *miR-377* and *Cpsf6* in L929 cells after transfection with mimic miR377. (**N**) Immunoblot analyses the expression of CPSF6 in L929 cells after transfection with mimic miR377. (**O** and **P**) qRT–PCR analyses of the mRNA abundance of *Cpsf6* in L929 cells infected with VSV-eGFP (**O**) or transfected with Poly (I:C) (**P**) for 6 h, followed by treatment with Act D (5 μg/mL) for the indicated times. Data are representative of three independent experiments, with one representative shown in (**N**). The values represent mean ± SD with individual measurements overlaid as dots, statistical analysis was performed using **Mann–Whitney U test in** (**A**-**E**) **or two-tailed Student’s *t*-test in** (**F**), (**K**-**M**), (**O**) and (**P**).
(TIF)

S8 Fig Up-regulated CPSF6 expression is associated with poor survival of multiple cancers. (**A**) Human *CPSF6* expression levels in different tumor types from TCGA database were determined by TIMER (*P < 0.05, **P < 0.01, ***P < 0.001). (**B**-**F**) Kaplan-Meier analysis of overall survival and disease-free survival of ACC, KIRP, LUAD, MESO or PADD patients (data from GEPIA). (**G**) Human miR-377-3p expression levels in different tumor types from TCGA database were determined by CancerMIRNome (*P < 0.05, **P < 0.01, ***P < 0.001).
(TIF)

S1 Table Primer sequences used in this study. (DOCX)

S1 Raw Images Original scan images for Figs 1E–1H, 2A, 2D–2E, 2H, 2J, 6F–6I, S1A–S1B, S2G, S2I, S6E–S6I and S7N. (PDF)

S1 Values For Plots Numerical values used for plots and statistical analysis in Figs 1A–1D, 2B, 2C, 2F, 2I, 2L, 2M, 4C–4N, 5A–5E, 5G–5K, 5M–5Q, 6A–6E, 6J–6R, S2A–S2C, S2E–S2F, S2H, S4H–S4K, S6A–S6D, S7K–S7M and S7O–S7P. (XLSX)
