# Supplementary figures and images for: Downregulation of CPSF6 leads to global mRNA 3’ UTR shortening and enhanced antiviral immune responses
Source: PLoS Pathog. 2024 Feb 28;20(2):e1012061. doi: 10.1371/journal.ppat.1012061 (PMC10927093; doi:10.1371/journal.ppat.1012061)

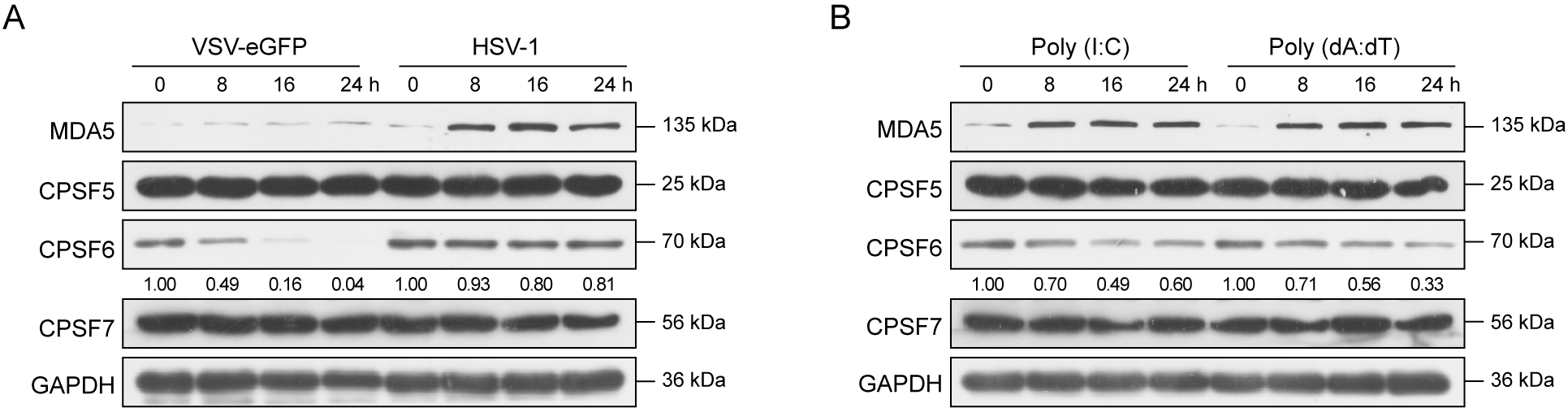

Supplement: S1 Fig — (A, B) Immunoblot analyses the CFIm complex expression in BMDMs infected with VSV-eGFP or HSV-1 (A) or stimulated with poly (I:C) or Poly (dA:dT) (B) at indicated time points. Data are representative of three independent experiments, with one representative shown in (A and B). (TIF) [file ppat.1012061.s001.tif]

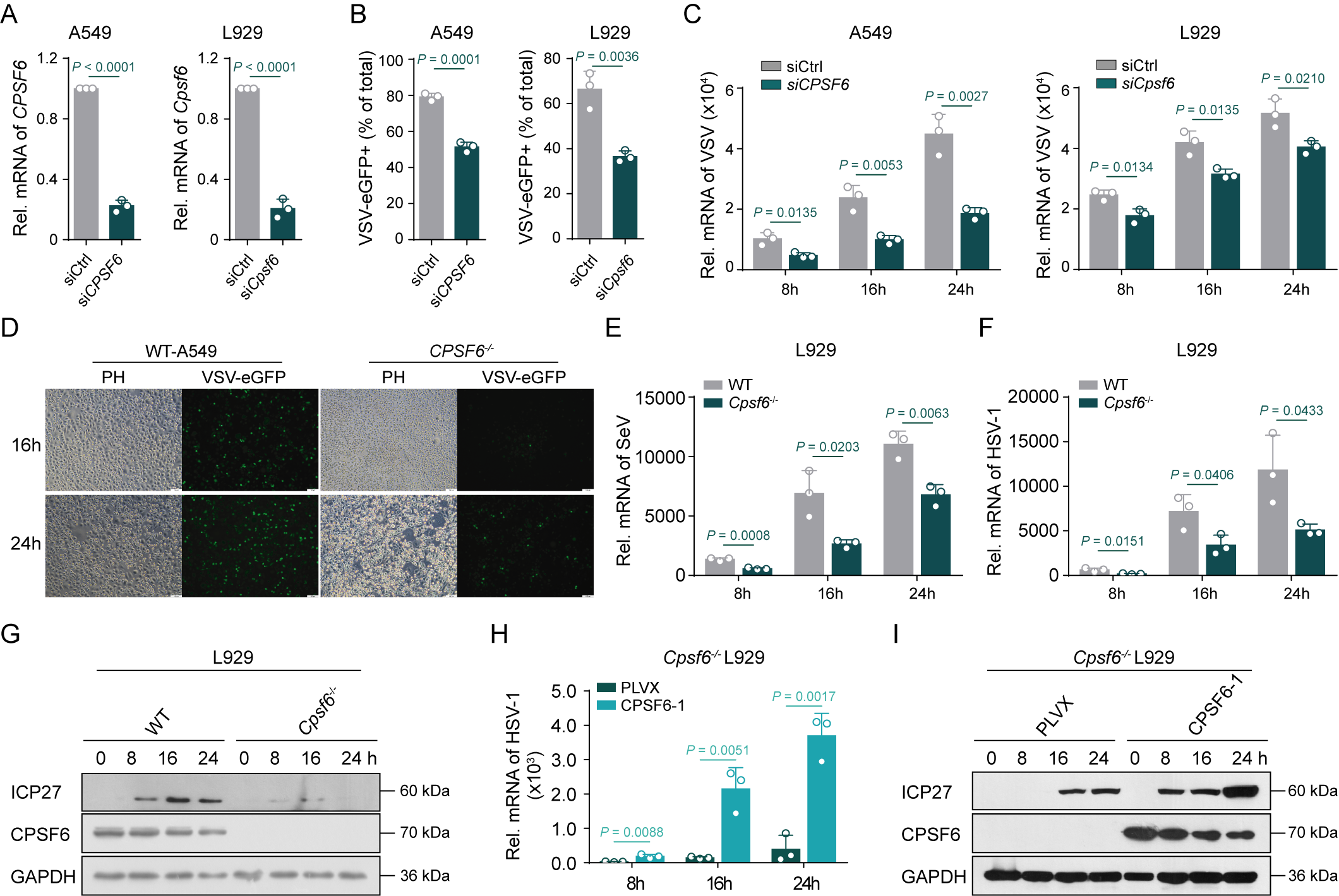

Supplement: S2 Fig — (A) qRT–PCR analyses of the knockdown efficiency of CPSF6 in A549 and L929 cells. (B) FACS analyses of VSV replication in CPSF6 knocked down A549 or L929 cells infected with VSV-eGFP for 12h. (C) qRT–PCR analyses of VSV replication in CPSF6 knocked down A549 and L929 cells infected with VSV-eGFP at indicated time points. (D) Fluorescence microscope analyses of GFP intensity in WT and CPSF6-/- A549 cells infected with VSV-eGFP for 16 or 24 h. Scale bar, 100 μm. (E, F) qRT–PCR analyses of SeV (E) or HSV-1 (F) replication in WT and Cpsf6-/- L929 cells. (G) Immunoblot analyses of the ICP27 expression in WT and Cpsf6-/- L929 cells infected with HSV-1 at indicated time points. (H) qRT–PCR analyses of HSV-1 replication in Cpsf6-/-L929PLVX and Cpsf6-/- L929CPSF6 L929 cells. (I) Immunoblot analyses of the ICP27 expression in Cpsf6-/-L929PLVX and Cpsf6-/- L929CPSF6 L929 cells infected with HSV-1 at indicated time points. Data are representative of three independent experiments, with one representative shown in (D), (G) and (I). The values represent mean ± SD with individual measurements overlaid as dots, statistical analysis was performed using a two-tailed Student’s t-test in (A-C), (E), (F) and (H). (TIF) [file ppat.1012061.s002.tif]

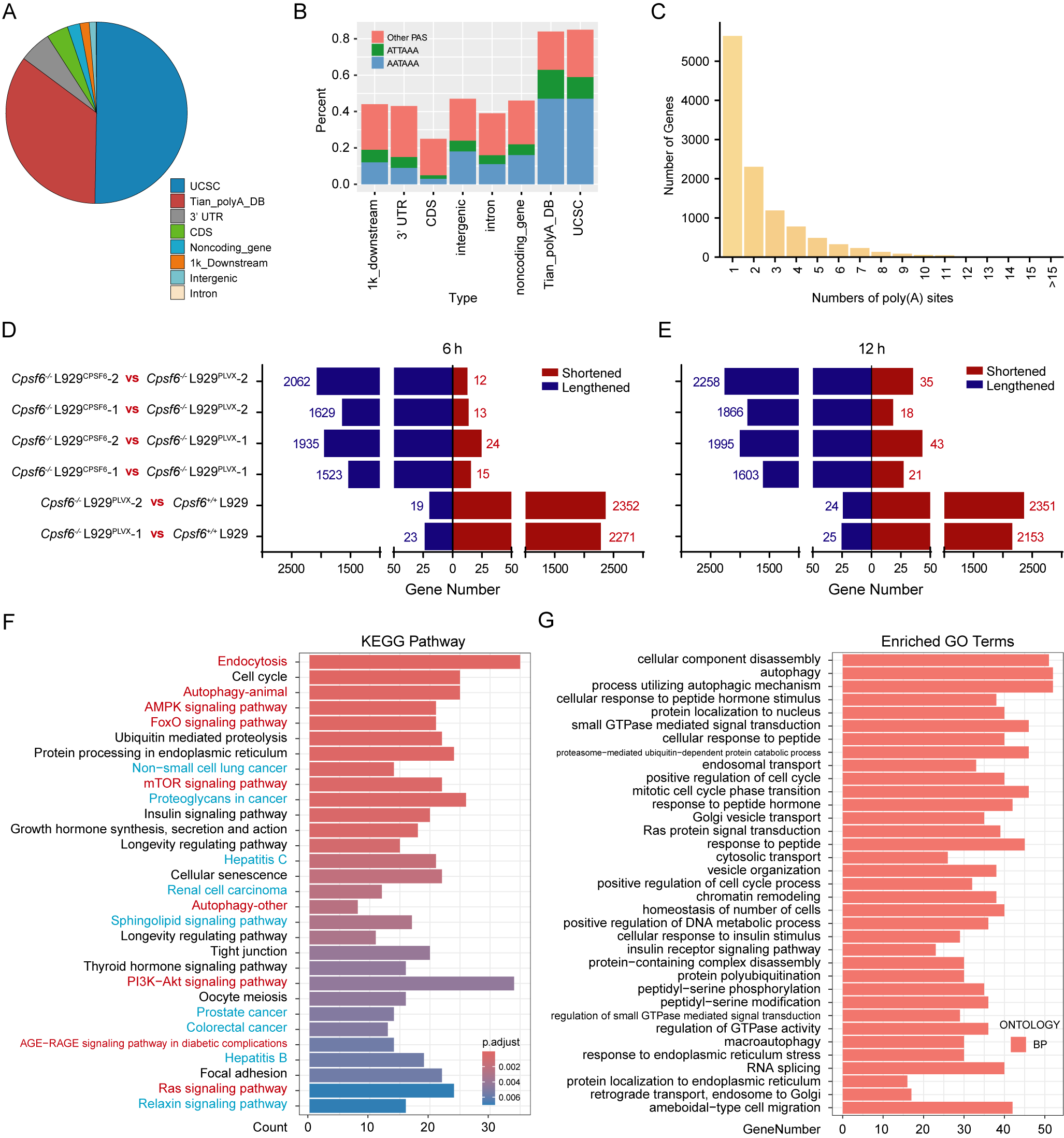

Supplement: S3 Fig — (A) Genomic locations of reads uniquely mapped to the nuclear genome after internal priming filtering. (B) Distribution of classical and non-classical poly(A) signal hexamer at different genomic locations. (C) Number of genes with different numbers of poly(A) sites. (D, E) The number of poly (A) site-switched genes in L929 cells infected with VSV-eGFP for 6 (D) or 12 h (E). (F, G) KEGG enrichment (F) and GO (G) analysis of the non-immune-related genes among the CPSF6 target genes. (TIF) [file ppat.1012061.s003.tif]

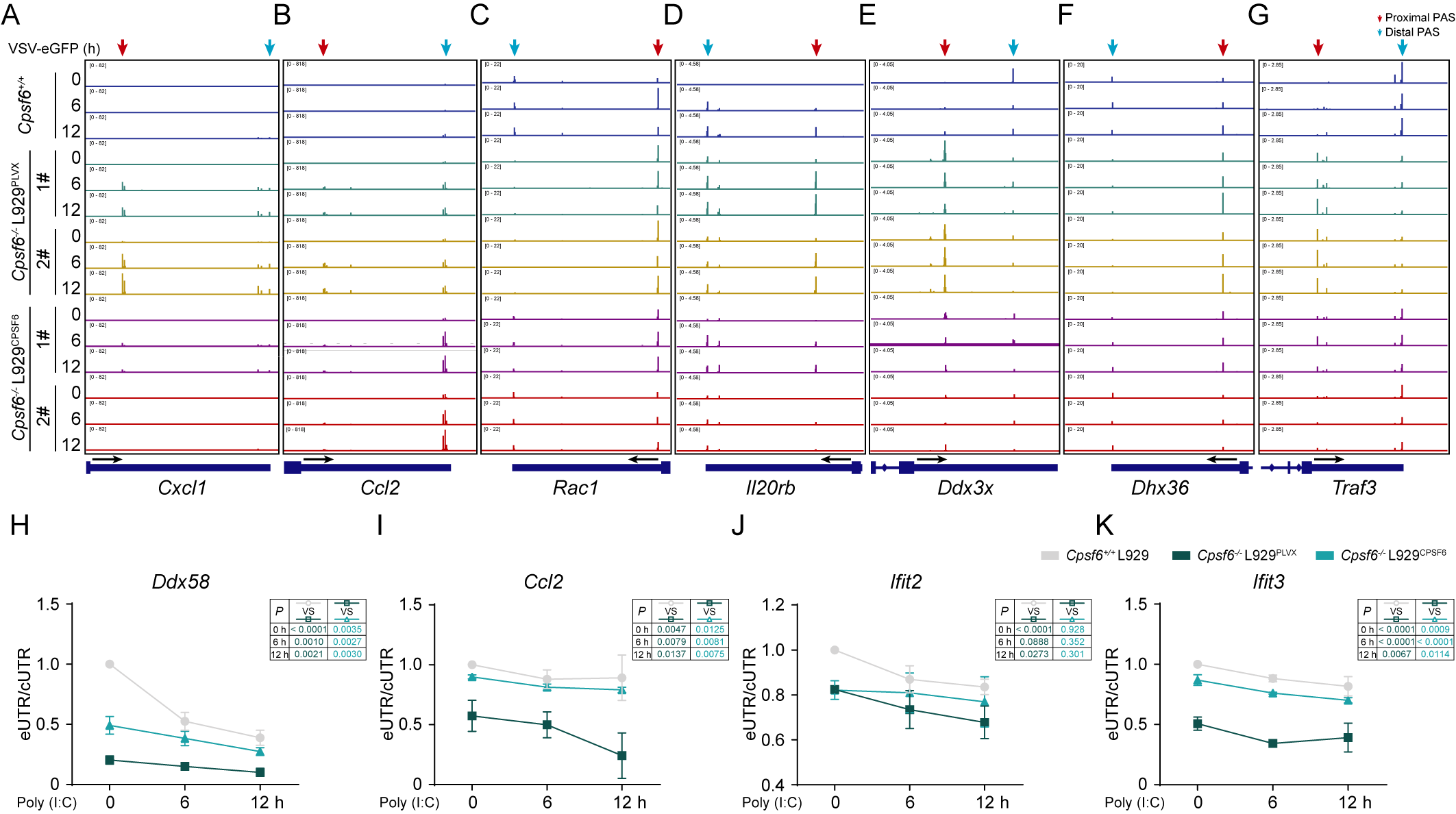

Supplement: S4 Fig — (A-G) IVT-SAPAS read alignments of Cxcl1 (A), Ccl2 (B), Rac1 (C), Il20rb (D), Ddx3x (E), Dhx36 (F) and Traf3 (G) are shown. (H-K) qRT–PCR analyses the ratio of longer 3’ UTR isoforms to total mRNA of tested genes Ddx58 (H), Ccl2 (I), Ifit2 (J) and Ifit3 (K) in WT, Cpsf6-/-L929PLVX and Cpsf6-/- L929CPSF6 L929 cells stimulated with poly (I:C) at indicated time points. (n = 3 replicates). The values represent mean ± SD with individual measurements overlaid as dots, statistical analysis was performed using a two-tailed Student’s t-test in (H-K). (TIF) [file ppat.1012061.s004.tif]

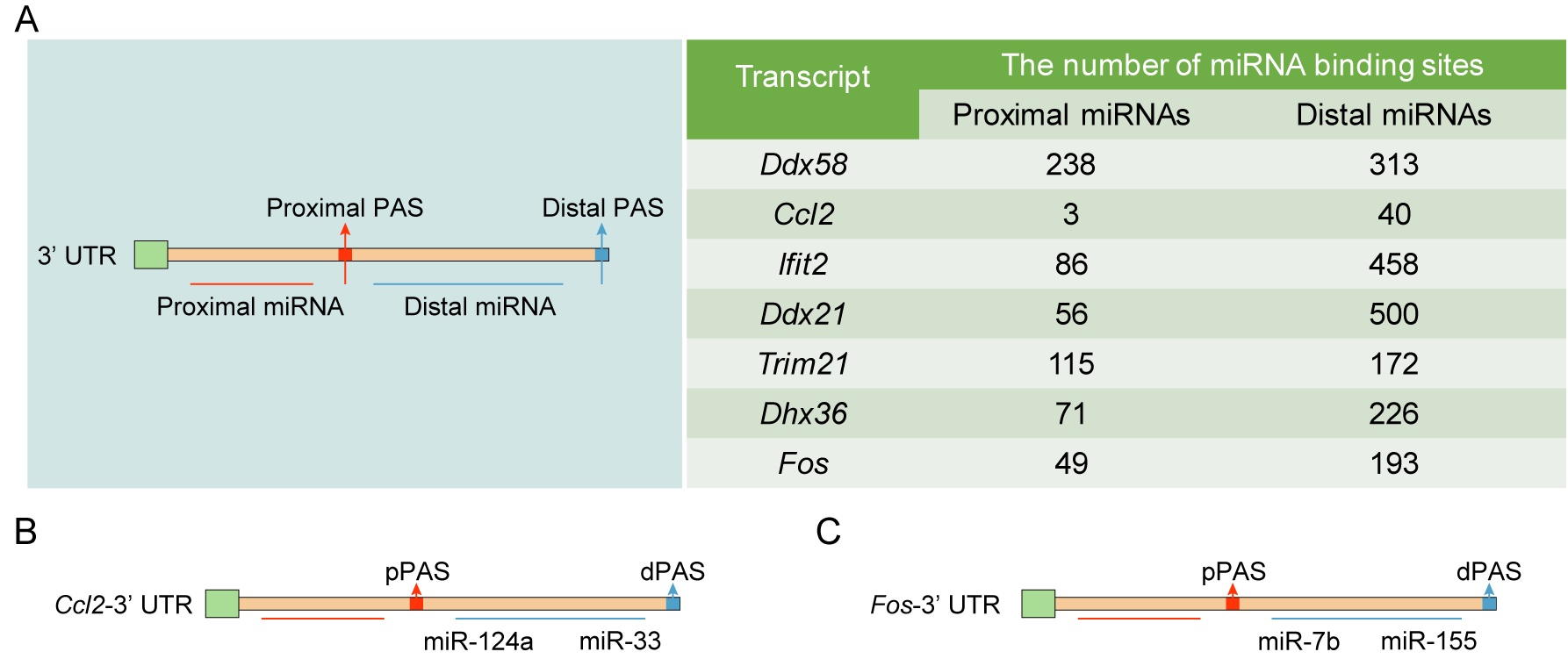

Supplement: S5 Fig — (A) The number of miRNAs binding to immune-related gene 3’ UTRs was predicted by a miRNA target prediction program (TargetScan). (B and C) Binding diagram of specific microRNAs in the 3’ UTR region of Ccl2 (B) and Fos (C) transcripts. (TIF) [file ppat.1012061.s005.tif]

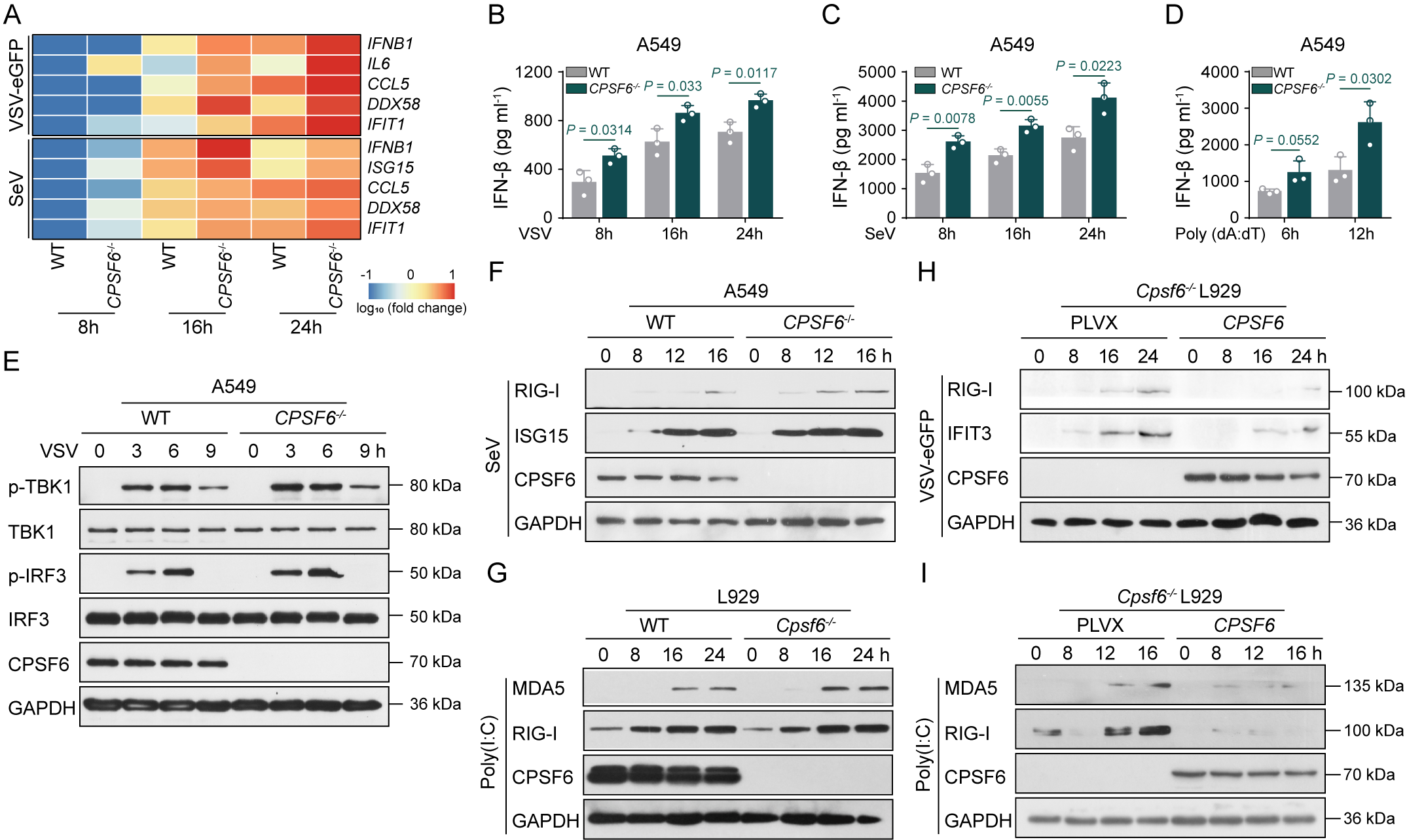

Supplement: S6 Fig — (A) The heatmap shows the mRNA abundance of IFNB and ISGs in WT and CPSF6-/- A549 cells infected with VSV-eGFP or SeV at indicated time points. (B-D) ELISA analyses the secretion of INF-β in cell supernatant from WT and CPSF6-/- A549 cells infected with VSV-eGFP (B) or SeV (C) or stimulated with Poly (dA:dT) (D) at indicated time points. (E) Immunoblot analyses of phosphorylated (p-) TBK1 and IRF3 in WT and CPSF6-/- A549 cells infected with VSV-eGFP at indicated time points. (F) Immunoblot analyses of the protein expression of ISGs in WT and CPSF6-/- A549 cells infected with SeV at indicated time points. (G) Immunoblot analyses of the protein expression of ISGs in WT and Cpsf6-/- L929 cells stimulated with Poly (I:C) at indicated time points. (H, I) Immunoblot analyses of the protein expression of ISGs in Cpsf6-/- L929PLVX and Cpsf6-/- L929CPSF6 L929 cells infected with VSV-eGFP (H) or stimulated with Poly (I:C) (I) at indicated time points. Data are representative of three independent experiments, with one representative shown in (E-I). The values represent mean ± SD with individual measurements overlaid as dots, statistical analysis was performed using a two-tailed Student’s t-test in (B-D). (TIF) [file ppat.1012061.s006.tif]

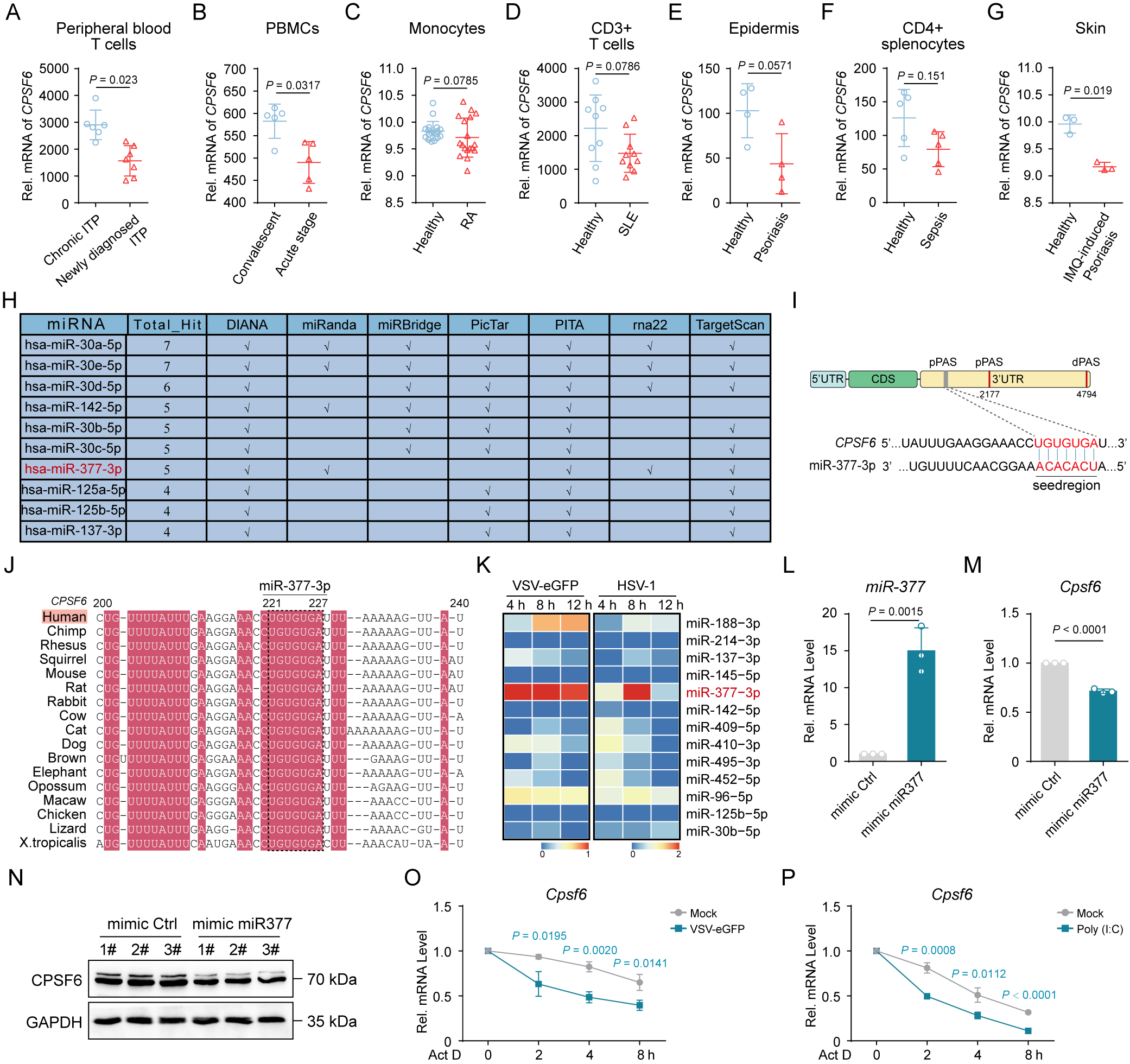

Supplement: S7 Fig — (A-G) The relative mRNA expression of CPSF6 in peripheral blood-T cells from ITP (A), PBMCs from asthma (B), monocytes from patients with RA (C), CD3+ T cells from patients with SLE (D), epidermis from psoriasis (E), CD4+ splenocytes from CLP-induced sepsis (F) and skin from IMQ-induced psoriasis (G) are shown. (H) The prediction of potential binding miRNAs on CPSF6 mRNA using the miRSystem database. (I) The schematic diagram of the binding of miR-377-3p and CPSF6-3’UTR. (J) Multiple sequence alignment of the CPSF6-3’ UTR showed the conservation of seedregion in tetrapods. (K) qRT–PCR analyses the expression of predicted miRNAs in L929 cells infected with VSV-eGFP or HSV-1 for the indicated times. (L and M) qRT–PCR analyses the expression of miR-377 and Cpsf6 in L929 cells after transfection with mimic miR377. (N) Immunoblot analyses the expression of CPSF6 in L929 cells after transfection with mimic miR377. (O and P) qRT–PCR analyses of the mRNA abundance of Cpsf6 in L929 cells infected with VSV-eGFP (O) or transfected with Poly (I:C) (P) for 6 h, followed by treatment with Act D (5 μg/mL) for the indicated times. Data are representative of three independent experiments, with one representative shown in (N). The values represent mean ± SD with individual measurements overlaid as dots, statistical analysis was performed using Mann–Whitney U test in (A-E) or two-tailed Student’s t-test in (F), (K-M), (O) and (P). (TIF) [file ppat.1012061.s007.tif]

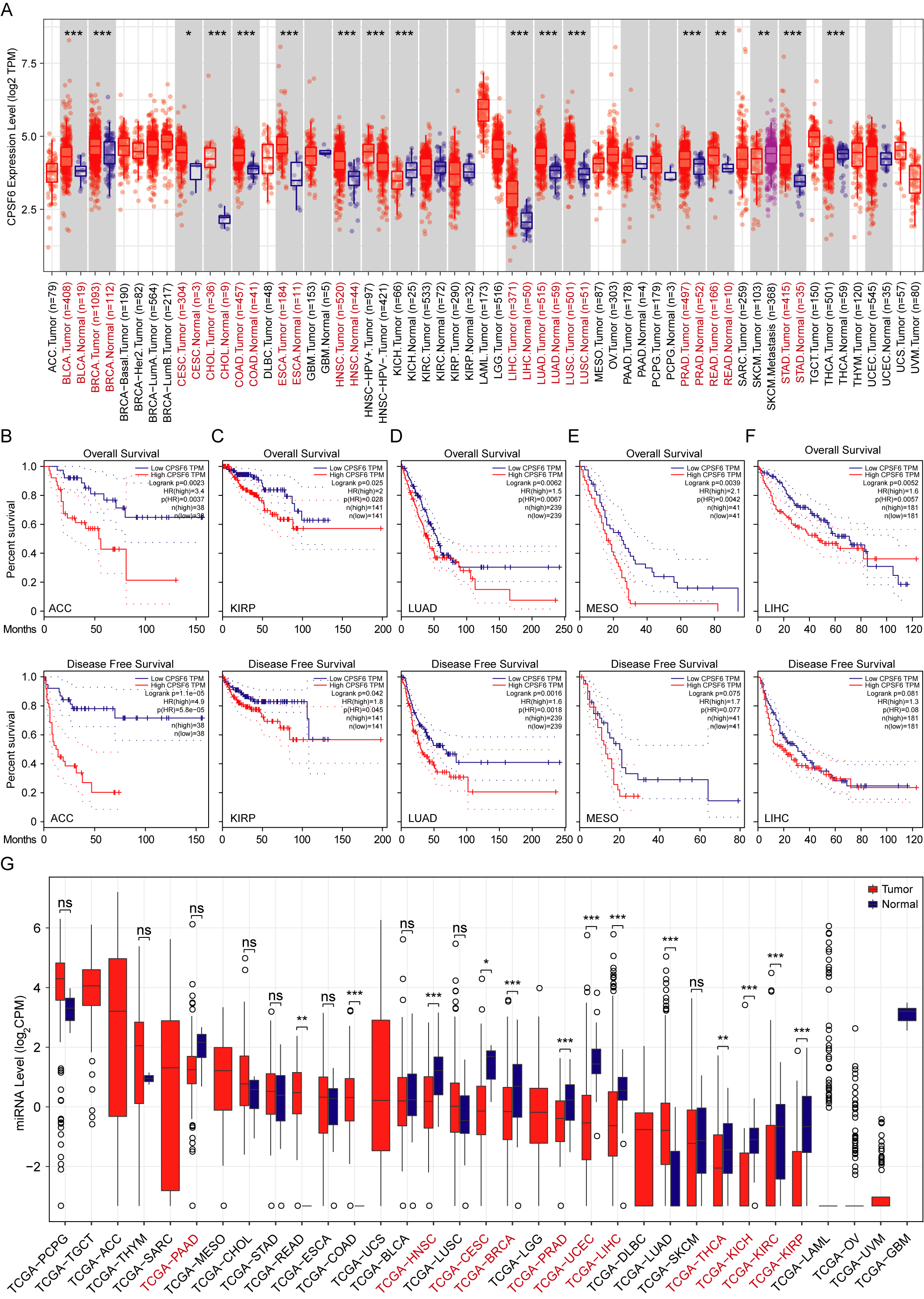

Supplement: S8 Fig — (A) Human CPSF6 expression levels in different tumor types from TCGA database were determined by TIMER (*P < 0.05, **P < 0.01, ***P < 0.001). (B-F) Kaplan-Meier analysis of overall survival and disease-free survival of ACC, KIRP, LUAD, MESO or PADD patients (data from GEPIA). (G) Human miR-377-3p expression levels in different tumor types from TCGA database were determined by CancerMIRNome (*P < 0.05, **P < 0.01, ***P < 0.001). (TIF) [file ppat.1012061.s008.tif]

Fig 1

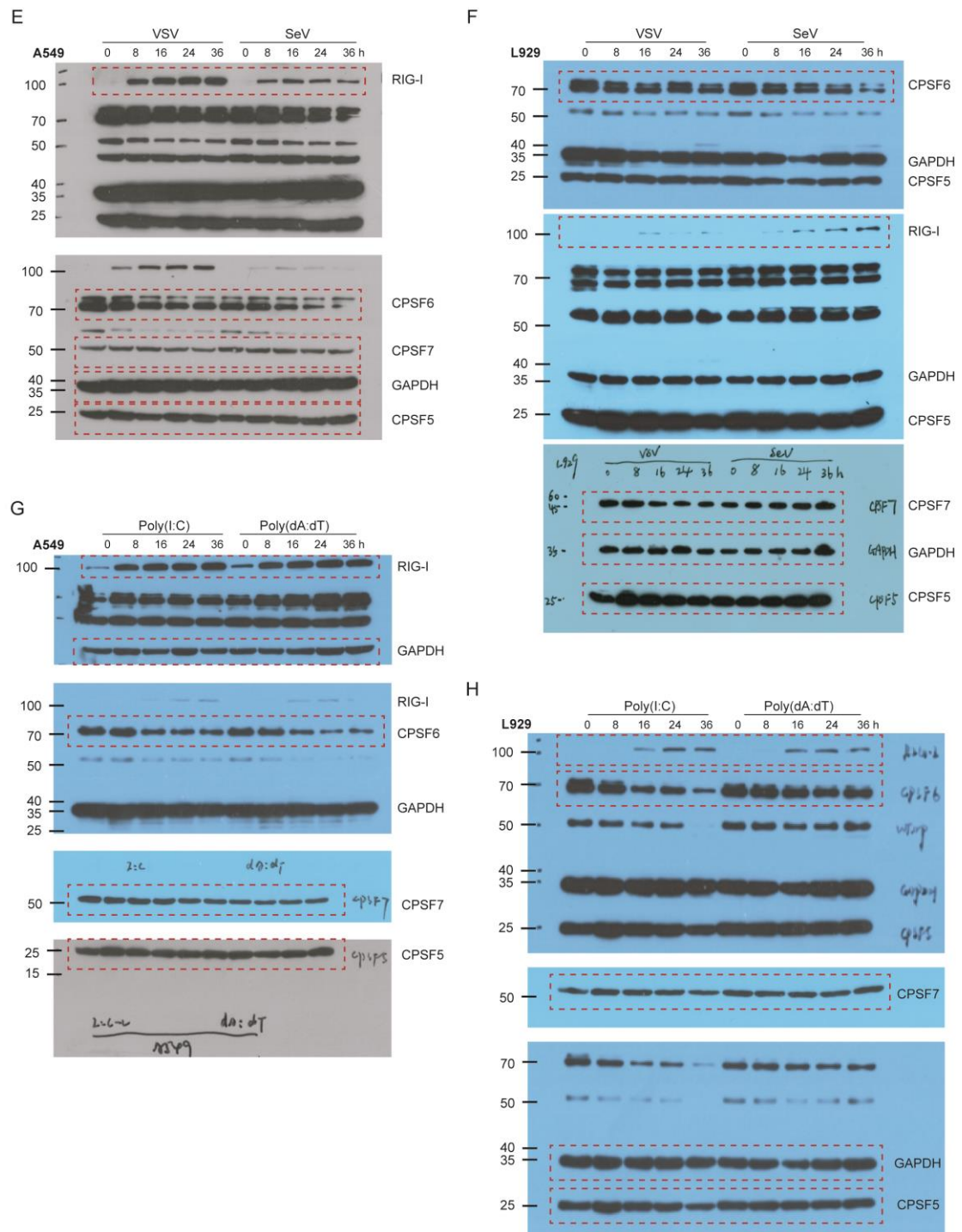

Fig 2

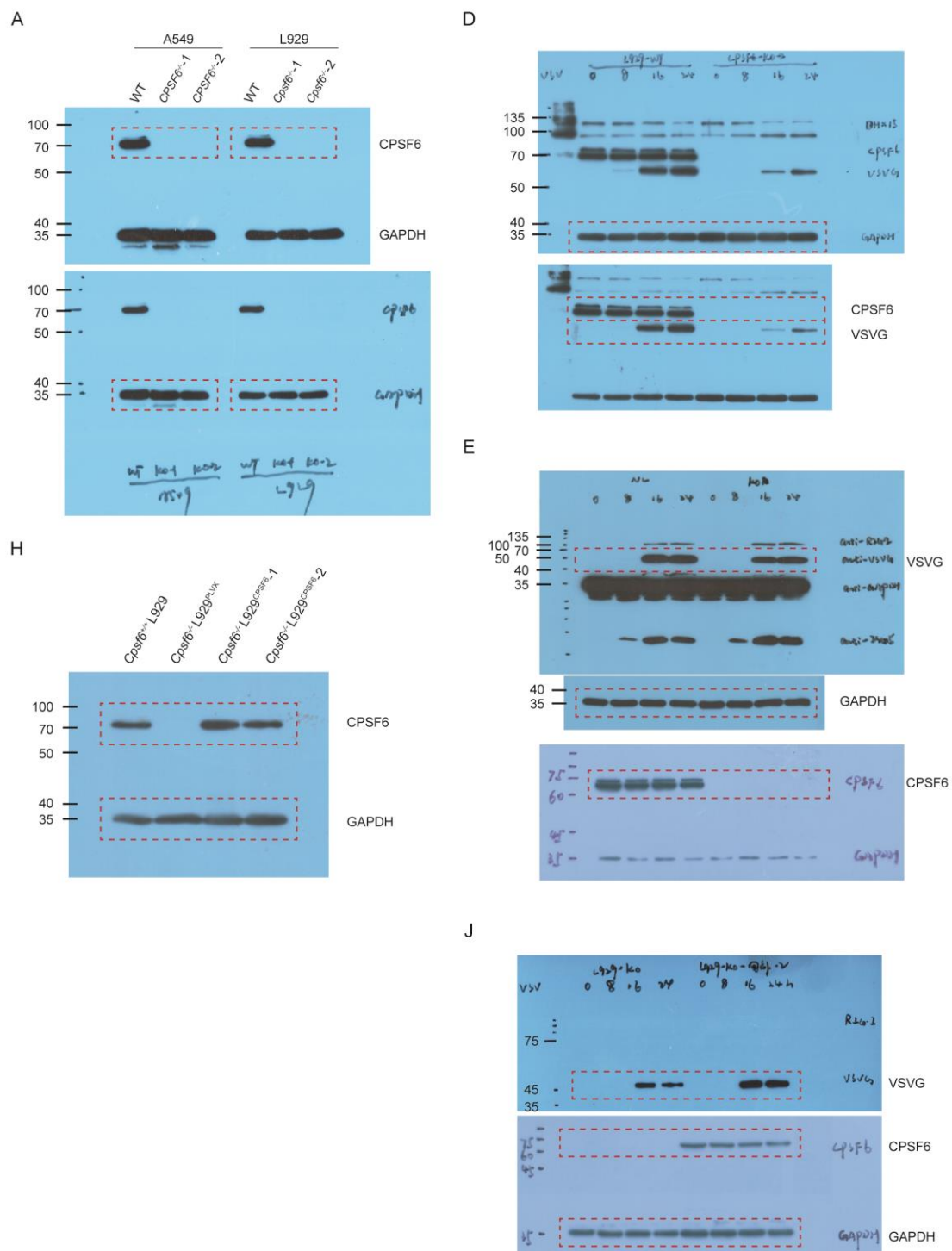

Fig 6

F

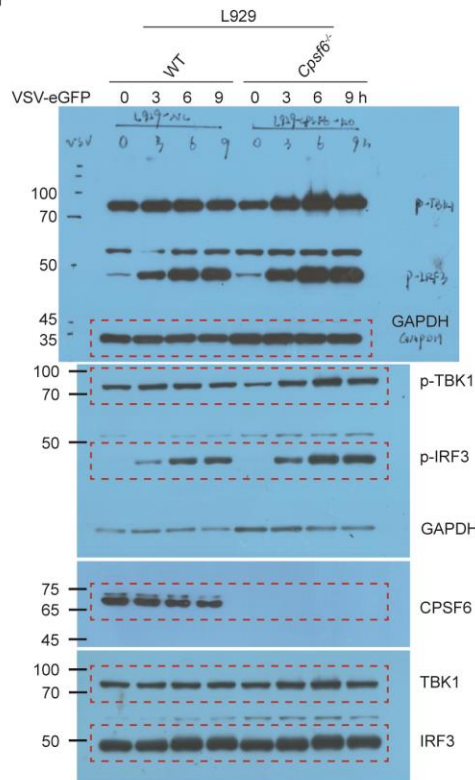

G

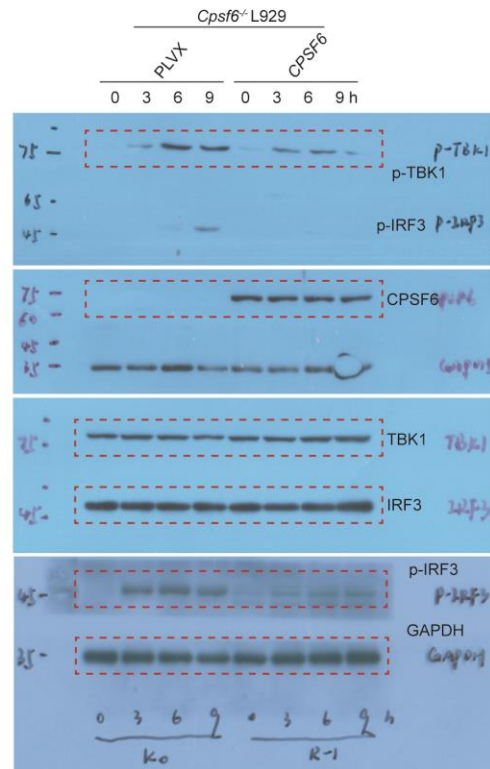

H

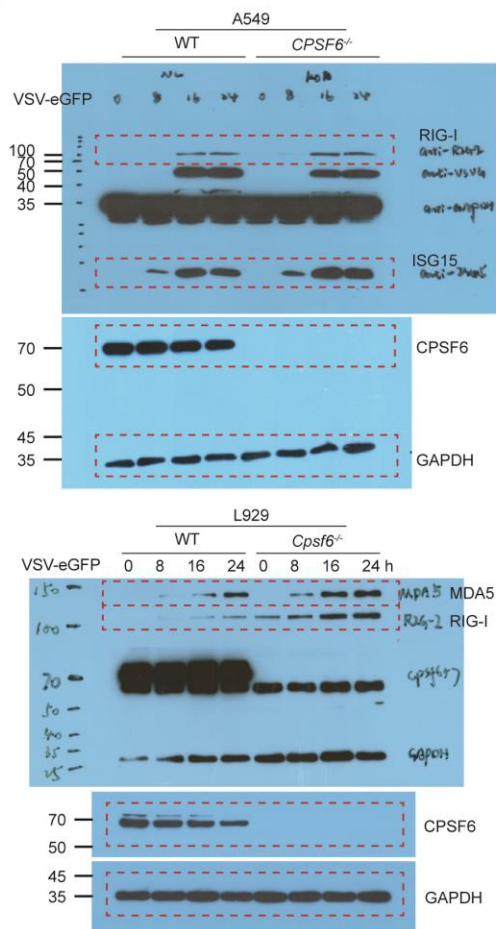

I

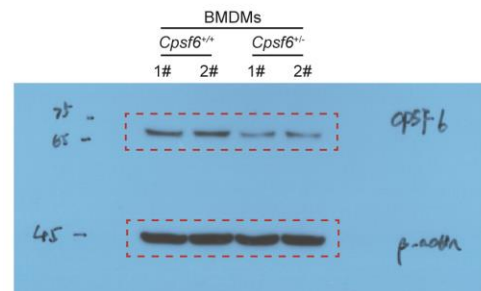

A

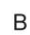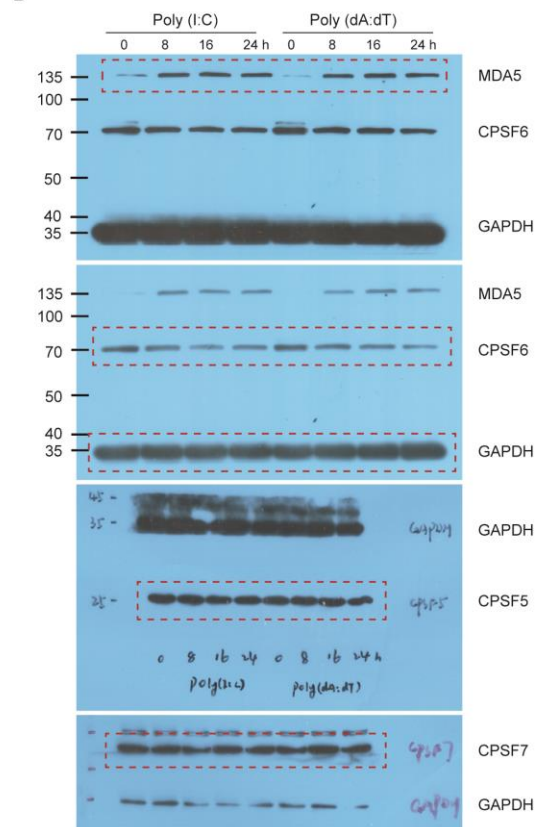

S2 Fig

G

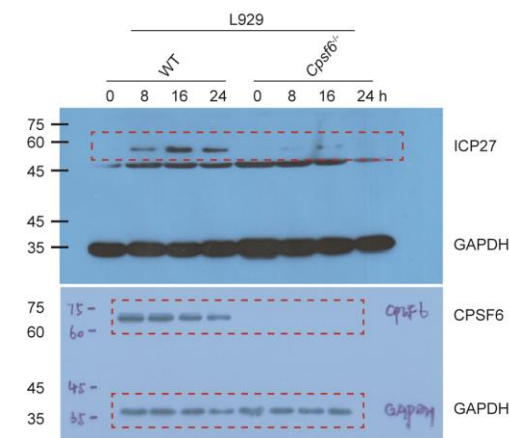

I

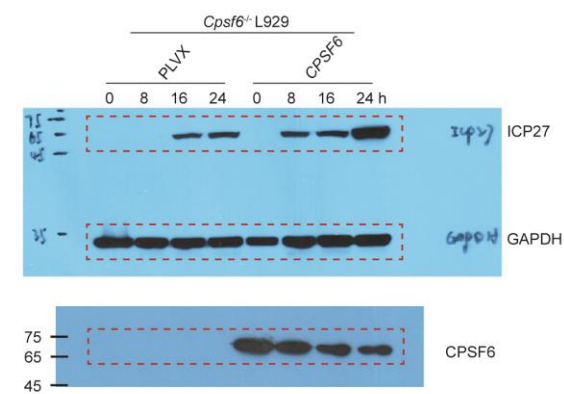

S6 Fig

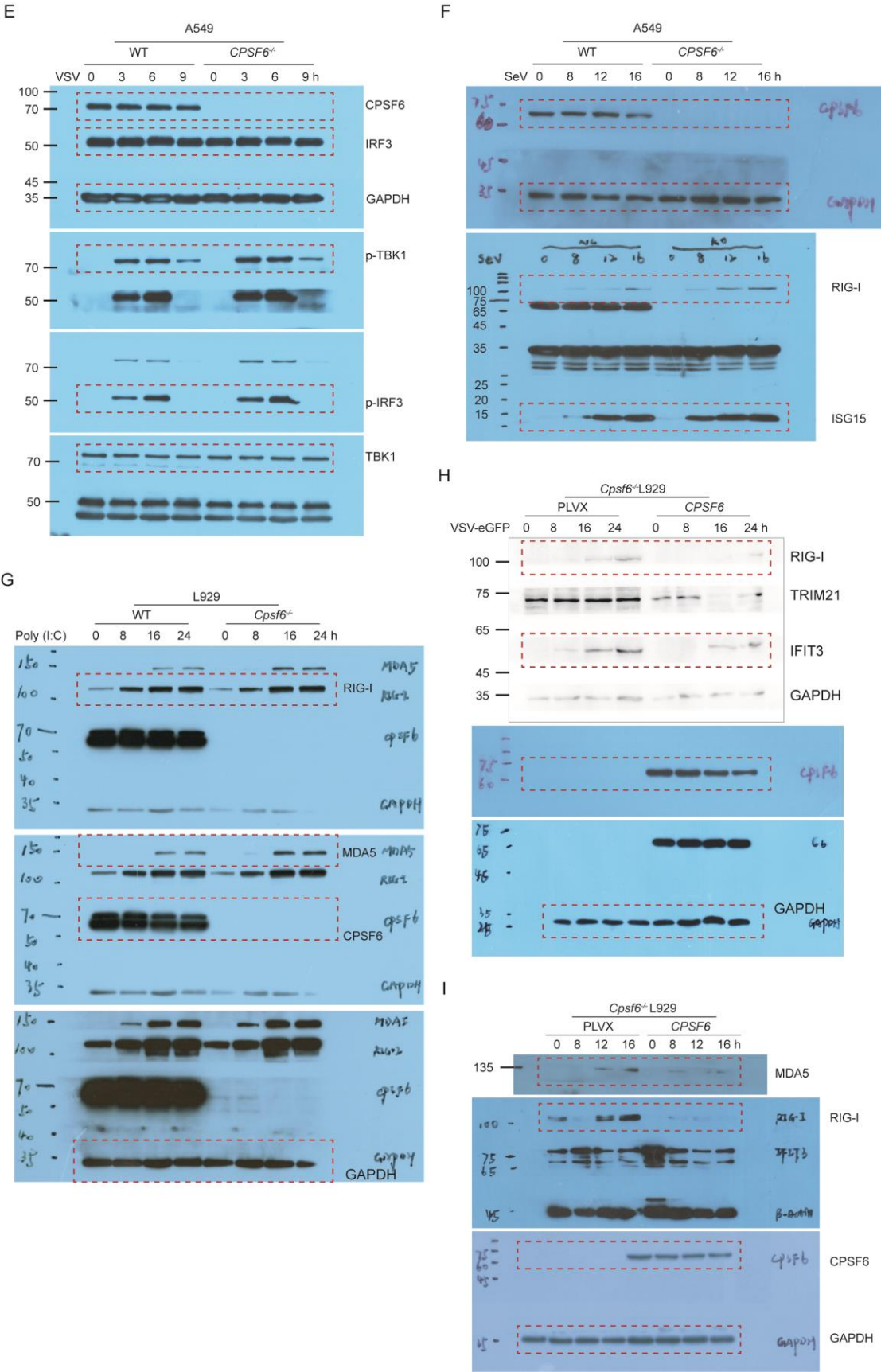

S7 Fig

N

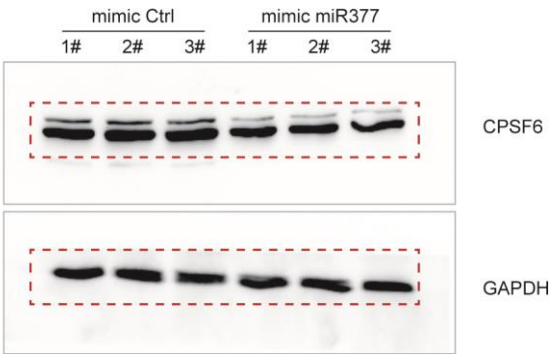

Supplement: S1 Raw Images — (PDF) [file ppat.1012061.s010.pdf]
