# Supplementary material for: Downregulation of CPSF6 leads to global mRNA 3’ UTR shortening and enhanced antiviral immune responses
Source: PLoS Pathog. 2024 Feb 28;20(2):e1012061. doi: 10.1371/journal.ppat.1012061 (PMC10927093; doi:10.1371/journal.ppat.1012061)
Supplement: S1 Table — (DOCX) [file ppat.1012061.s009.docx]

**S1 Table. Primer sequences used in this study.**

| **Primer** | **Forward sequence (5’ to 3’)** | **Reverse sequence (5’ to 3’)** |
| --- | --- | --- |
| *Cpsf5* | GTCAACCAGTTCGGCAACAAG | AGCTGTCCTTCTCATAGAGGG |
| *Cpsf6* | CATTTACGCGGATGTGGGTGA | CGCCATTATTTGCAGATGGAGA |
| *Cpsf7* | CGAGTCCCAAGAGGGGGAATA | GAGGGGGCATGAGATGCTG |
| *Ifnb1* | AGCTCCAAGAAAGGACGAACA | GCCCTGTAGGTGAGGTTGAT |
| *Il6* | CTGCAAGAGACTTCCATCCAG | AGTGGTATAGACAGGTCTGTTGG |
| *Ccl5* | TTTGCCTACCTCTCCCTCG | CGACTGCAAGATTGGAGCACT |
| *Isg15* | GGTGTCCGTGACTAACTCCAT | CTGTACCACTAGCATCACTGTG |
| *Ifit2* | AGTACAACGAGTAAGGAGTCACT | AGGCCAGTATGTTGCACATGG |
| *Aim2* | GTCACCAGTTCCTCAGTTGTG | CACCTCCATTGTCCCTGTTTTAT |
| *Ifit3* | TCAGGCTTACGTTGACAAGGT | CACACTTTAGGCGTGTCCATC |
| *Ifit3*-L-3’UTR | AGCAGACAGAGCACCTTTTGA | GCAAAGGTTAATCCTTCAGGC |
| *Ccl2* | TTAAAAACCTGGATCGGAACCAA | GCATTAGCTTCAGATTTACGGGT |
| *Ccl2*-L-3’UTR | TGCAAGGTGTGGATCCATTT | CCTTGGAATCTCAAACACAAAGT |
| *Ddx58* | GAAGAGCCAGAGTGTCAGAATC | AGCTCCAGTTGGTAATTTCTTGG |
| *Ddx58*-L-3’UTR | CAACCCTGGAGGTCAACAGT | GTTGCTGGGTAATTTCTCTAGGC |
| *Ddx21* | GAGGCCGTTTCCTCCAAAG | GTAGAAGCAGTGTCGTCTTGAG |
| *Ddx21*-L-3’UTR | CAGCTCAGAGAGCCCTTCTA | TCAAGCTTCTGTGCTAGGCG |
| *Ddx3x* | GTAGAAGCAGTGTCGTCTTGAG | TCACCCCGTGATCCAAAACTG |
| *Ddx3x*-L-3’UTR | CCGCCTACCCCTATCCCAAA | ACCTGTGTGCCAAGGTTTGA |
| *Dhx36* | TCACCCCGTGATCCAAAACTG | ATACCCATGATCCTCAGGAGC |
| *Dhx36*-L-3’UTR | TGGTCTTCACACAGTGCTTG | GCATAAAGAAGCATAAAAATGGT |
| *Trim21* | GGGAGGAGGTCACCTGTTCTA | GGCACTCGGGACATGAACTG |
| *Trim21*-L-3’UTR | TCCCCAGTGATAGCCAAAGT | TATCCACAAAATGACAGCAGGC |
| *Actb* | GTGACGTTGACATCCGTAAAGA | GCCGGACTCATCGTACTCC |
| *Gapdh* | TGACCTCAACTACATGGTCTACA | CTTCCCATTCTCGGCCTTG |
| *CPSF6* | GCATTTCCACAAGGTGGTAGA | GAGGGCGATCTCCTCGATTAG |
| *IFNB1* | GCTTGGATTCCTACAAAGAAGCA | ATAGATGGTCAATGCGGCGTC |
| *IL6* | ACTCACCTCTTCAGAACGAATTG | CCATCTTTGGAAGGTTCAGGTTG |
| *CCL5* | CCAGCAGTCGTCTTTGTCAC | CTCTGGGTTGGCACACACTT |
| *DDX58* | TGTGCTCCTACAGGTTGTGGA | CACTGGGATCTGATTCGCAAAA |
| *IFIT1* | AGAAGCAGGCAATCACAGAAAA | CTGAAACCGACCATAGTGGAAAT |
| *ACTB* | CATGTACGTTGCTATCCAGGC | CTCCTTAATGTCACGCACGAT |
| GAPDH | TGTTGCCATCAATGACCCCTT | CTCCACGACGTACTCAGCG |
| VSVG | CAAGTCAAAATGCCCAAGAGTCACA | TTTCCTTGCATTGTTCTACAGATGG |
| SeV | GCTTACGGGACAGATGAGAT | ATTGTTATGAACCGACTTGC |
| ICP27 | TTTCTCCAGTGCTACCTGAAGG | TCAACTCGCAGACACGACTCG |
| miR-188-3p | CATCCCTTGCATGGTGGAGGG |  |
| miR-214-3p | ACAGCAGGCACAGACAGGCAGT |  |
| miR-137-3p | TTATTGCTTAAGAATACGCGTAG |  |
| miR-145-5p | GTCCAGTTTTCCCAGGAATCCCT |  |
| miR-377-3p | ATCACACAAAGGCAACTTTTGT |  |
| miR-142-5p | CATAAAGTAGAAAGCACTACT |  |
| miR-409-5p | AGGTTACCCGAGCAACTTTGCAT |  |
| miR-410-3p | AATATAACACAGATGGCCTGT |  |
| miR-495-3p | AAACAAACATGGTGCACTTCTT |  |
| miR-452-5p | AACTGTTTGCAGAGGAAACTGA |  |
| miR-96-5p | TTTGGCACTAGCACATTTTTGCT |  |
| miR-125b-5p | TCCCTGAGACCCTAACTTGTGA |  |
| miR-30b-5p | TGTAAACATCCTACACTCAGCT |  |
